# Supplementary material for: Rehabilitation outcomes following tail-fluke amputation in an Indo-Pacific bottlenose dolphin: A welfare-centered approach
Source: PLoS One. 2026 Mar 13;21(3):e0331642. doi: 10.1371/journal.pone.0331642 (PMC12987451; doi:10.1371/journal.pone.0331642)
Supplement: Attachment — Submitted filename: PONE-D-25-45185_R1 (1).pdf [file pone.0331642.s009.pdf]

PONE-D-25-45185_R1.pdf

2025-11

2025-11

Instituto Politecnico Nacional

Detalles del documento

Identificador de la entrega

trn:oid:::14652:533329514

Fecha de entrega

26 nov 2025, 2:11 p.m. GMT-6

Fecha de descarga

26 nov 2025, 2:17 p.m. GMT-6

Nombre del archivo

PONE-D-25-45185_R1.pdf

Tamaño del archivo

7.2 MB

94 páginas

18.764 palabras

110.260 caracteres

77 % detectado como IA

El porcentaje indica la cantidad combinada del texto generado probablemente con IA, así como el texto generado con IA que también se parafraseó probablemente con IA.

Precaución: Se necesita revisión.

Es esencial comprender los límites de la detección de IA antes de tomar decisiones acerca del trabajo del estudiante. Te alentamos a obtener más información acerca de las funciones de detección de IA de Turnitin antes de usar la herramienta.

Grupos de detección

116 Sólo generado con IA 77%
Texto generado probablemente con IA desde un modelo de lenguaje de gran tamaño.

0 Texto generado con IA que se parafraseó con IA 0%
Texto generado probablemente con IA que se revisó probablemente usando una herramienta de parafraseo de IA o de reescritura.

Aviso legal
Nuestra evaluación de escritura con IA está diseñada para ayudar a los académicos a identificar texto que podrían haberse preparado mediante una herramienta de IA generativa. Es posible que nuestra evaluación de escritura con IA no siempre sea precisa (por ejemplo, nuestros modelos de IA pueden producir tanto resultados falsos positivos como falsos negativos), por lo que no debe usarse como único fundamento para aplicar sanciones a un estudiante. Para determinar si es un caso de deshonestidad académica, se necesita de un escrutinio mayor y el juicio humano, junto con la aplicación de las políticas académicas específicas de la organización.

Preguntas frecuentes

¿Cómo debería interpretar los falsos positivos y el porcentaje de escritura con IA de Turnitin?
El porcentaje que se muestra en el reporte de escritura con IA es la cantidad del texto calificado en la entrega que el modelo de detección de escritura con IA de Turnitin determina se generó probablemente con IA desde un modelo de lenguaje de gran tamaño, o se generó probablemente con IA y es probable que se haya revisado usando una herramienta de parafraseo de IA o una herramienta aleatoria de palabras.

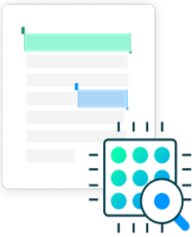

Los falsos positivos (que marcan incorrectamente alertas de texto escrito por humanos como generado con IA) son una posibilidad en los modelos de IA.

Los puntajes de detección de IA inferiores al 20 %, que no aparecen en reportes nuevos, tienen una mayor probabilidad de ser falsos positivos. Para reducir la probabilidad de malinterpretación, no se atribuye ningún puntaje o resaltado y se indican con un asterisco en el reporte (*%).

El porcentaje de escritura con IA no debe ser el único fundamento para determinar si ha ocurrido una mala conducta. El revisor/instructor debería usar el porcentaje como un medio para iniciar una conversación formativa con sus estudiantes o usarlo para examinar el ejercicio entregado según las políticas de la escuela.

¿Qué significa 'texto calificado'?
Nuestro modelo sólo procesa texto calificado en la forma de escritura de formato largo. La escritura de formato largo significa que los enunciados individuales en párrafos que constituyen una parte más grande del trabajo escrito, como un ensayo, una disertación, un artículo, etc. El texto calificado que se ha determinado que se generó probablemente con IA se resaltará en color cian en la entrega, y el texto generado probablemente con IA y luego parafraseado probablemente con IA se resaltará en color morado.

El texto no calificado, como viñetas, bibliografías comentadas, etc., no se procesará y puede crear disparidad entre los puntos destacados de la entrega y el porcentaje mostrado.

PLOS ONE

Rehabilitation outcomes following tail-fluke amputation in an Indo-Pacific bottlenose dolphin: a welfare-centered approach

--Manuscript Draft--

**Manuscript Number:**	PONE-D-25-45185R1
**Article Type:**	Research Article
**Full Title:**	Rehabilitation outcomes following tail-fluke amputation in an Indo-Pacific bottlenose dolphin: a welfare-centered approach
**Short Title:**	Welfare-centered rehabilitation after tail-fluke amputation in a captive Indo-Pacific bottlenose dolphin
**Corresponding Author:**	Shin-ichiro Oka<br>Okinawa Churashima Foundation<br>Motobu-cho, Okinawa JAPAN
**Keywords:**	animal rehabilitation; prosthetic device; Animal welfare; swimming performance; social behavior; behavioral adaptation; dolphin psychology; Indo-Pacific bottlenose dolphin; marine mammal care
**Abstract:**	Tail fluke loss in cetaceans compromises locomotion and impairs social functioning, posing serious welfare challenges. Rehabilitation strategies that address both physical performance and behavioral reintegration are essential for improving quality of life in affected individuals. This study evaluated the outcomes of a structured, two-phase rehabilitation program applied to Sami, an adult Indo-Pacific bottlenose dolphin (Tursiops aduncus) that underwent tail-fluke amputation, with an emphasis on locomotor function, behavioral adaptation, and long-term welfare. The rehabilitation protocol comprised two phases. Phase 1 focused on restoring vertical tail-beat locomotion through a combination of range-of-motion exercises and the use of a custom-designed prosthetic tail fluke. Swimming performance was quantitatively assessed using biologging devices under three conditions: without tail flukes, with the prosthetic tail, and in healthy conspecifics. Phase 2 introduced structured cohabitation with familiar individuals to promote social reintegration. Behavioral data were collected before and after rehabilitation to evaluate affiliative engagement and activity patterns. While maximum swim speed remained lower than in healthy individuals, propulsion per stroke significantly improved with prosthetic use. Notably, the subject dolphin retained species-typical vertical tail-beat motion even after prosthesis discontinuation, indicating motor pattern adaptation. Post-rehabilitation, affiliative behavior increased to 17% of total observed activity—more than twice that of a healthy control—while resting behavior markedly declined. No aggression or abnormal behaviors were observed. This study demonstrates that a welfare-centered, multi-phase rehabilitation framework can effectively promote both functional recovery and social reengagement in dolphins with severe caudal injuries. The long-term retention of adaptive locomotor and social behaviors highlights the potential of integrative approaches to enhance the quality of life in physically compromised cetaceans.
**Order of Authors:**	Suguru Higa
	Sayaka Takahashi
	Eri Nakashima
	Yui Kurosu
	Haruka Ikeshima
	Ryota Yagi
	Nihiro Adachi
	Keiichi Ueda
	Hirobumi Umeyama
	Hitoshi Yamamoto

Powered by Editorial Manager® and ProduXion Manager® from Aries Systems Corporation

	Yukinori Nakakita
	Kazuma Tochigi
	Taihei Kagawa
	Shin-ichiro Oka, PhD
Response to Reviewers:	Thank you very much for the opportunity to revise our manuscript. We sincerely appreciate the constructive comments provided by you and the reviewers.<br>We have carefully revised the paper to address all feedback. Major improvements include the addition of recent literature (2020–2024), expanded methodological details, clarification of ethical decision-making, and enhancement of long-term welfare discussion and practical rehabilitation guidelines. A new supplementary video has also been added to illustrate the prosthesis fitting process.<br>A detailed point-by-point response is provided in the attached Response to Reviewers document. We believe these revisions have strengthened the scientific rigor, ethical transparency, and welfare relevance of the study.
Additional Information:	
Question	Response
**Financial Disclosure**<br><br>Enter a financial disclosure statement that describes the sources of funding for the work included in this submission. Review the submission guidelines for detailed requirements. View published research articles from *PLOS ONE* for specific examples.<br><br>This statement is required for submission and **will appear in the published article** if the submission is accepted. Please make sure it is accurate.<br><br>**Funded studies**<br>Enter a statement with the following details:<br>• Initials of the authors who received each award<br>• Grant numbers awarded to each author<br>• The full name of each funder<br>• URL of each funder website<br>• Did the sponsors or funders play any role in the study design, data collection and analysis, decision to publish, or preparation of the manuscript?<br><br>Did you receive funding for this work?	The author(s) received no specific funding for this work.
**Competing Interests**<br><br>Use the instructions below to enter a competing interest statement for this	The authors have declared that no competing interests exist.

Powered by Editorial Manager® and ProduXion Manager® from Aries Systems Corporation

submission. On behalf of all authors, disclose any competing interests that could be perceived to bias this work—acknowledging all financial support and any other relevant financial or non-financial competing interests.<br><br>This statement is required for submission and **will appear in the published article** if the submission is accepted. Please make sure it is accurate and that any funding sources listed in your Funding Information later in the submission form are also declared in your Financial Disclosure statement.<br><br>View published research articles from *PLOS ONE* for specific examples.<br><br>**NO authors have competing interests**<br><br>Enter: *The authors have declared that no competing interests exist.*<br><br>**Authors with competing interests**<br><br>Enter competing interest details beginning with this statement:<br><br>*I have read the journal's policy and the authors of this manuscript have the following competing interests: [insert competing interests here]*<br><br>* typeset	
**Ethics Statement**<br><br>Enter an ethics statement for this submission. This statement is required if the study involved:<br><br>• Human participants<br>• Human specimens or tissue<br>• Vertebrate animals or cephalopods<br>• Vertebrate embryos or tissues<br>• Field research<br><br>Write "N/A" if the submission does not	This study was conducted in accordance with the Animal Experimentation Guidelines of the Okinawa Churashima Foundation (Motobu, Okinawa, Japan). The experimental protocol was proposed by co-author Eri Nakashima and formally submitted on November 30, 2022.<br>The protocol was reviewed and approved by the Animal Experiment Committee of the Okinawa Churashima Foundation (Protocol Number: AT22003). The review process included examination of the proposed methodology, animal welfare considerations, and justification for the use of prosthetic devices to improve the quality of life (QOL) of dolphins with impaired swimming ability. All procedures were designed to minimize stress and avoid injury.<br>The committee convened between December 2 and December 19, 2022, and the proposal was officially authorized by the President of the Okinawa Churashima Foundation on January 4, 2023.

Powered by Editorial Manager® and ProduXion Manager® from Aries Systems Corporation

require an ethics statement.<br><br>General guidance is provided below. Consult the submission guidelines for detailed instructions. **Make sure that all information entered here is included in the Methods section of the manuscript.**<br><br>**Format for specific study types**<br><br>**Human Subject Research (involving human participants and/or tissue)**<br>• Give the name of the institutional review board or ethics committee that approved the study<br>• Include the approval number and/or a statement indicating approval of this research<br>• Indicate the form of consent obtained (written/oral) or the reason that consent was not obtained (e.g. the data were analyzed anonymously)<br><br>**Animal Research (involving vertebrate animals, embryos or tissues)**<br>• Provide the name of the Institutional Animal Care and Use Committee (IACUC) or other relevant ethics board that reviewed the study protocol, and indicate whether they approved this research or granted a formal waiver of ethical approval<br>• Include an approval number if one was obtained<br>• If the study involved *non-human primates*, add *additional details* about animal welfare and steps taken to ameliorate suffering<br>• If anesthesia, euthanasia, or any kind of animal sacrifice is part of the study, include briefly which substances and/or methods were applied<br><br>**Field Research**<br><br>Include the following details if this study involves the collection of plant, animal, or other materials from a natural setting:<br>• Field permit number<br>• Name of the institution or relevant body that granted permission	
**Data Availability**	Yes - all data are fully available without restriction

Powered by Editorial Manager® and ProduXion Manager® from Aries Systems Corporation

Authors are required to make all data underlying the findings described fully available, without restriction, and from the time of publication. PLOS allows rare exceptions to address legal and ethical concerns. See the PLOS Data Policy and FAQ for detailed information.<br><br>A Data Availability Statement describing where the data can be found is required at submission. Your answers to this question constitute the Data Availability Statement and **will be published in the article**, if accepted.<br><br>**Important:** Stating 'data available on request from the author' is not sufficient. If your data are only available upon request, select 'No' for the first question and explain your exceptional situation in the text box.<br><br>Do the authors confirm that all data underlying the findings described in their manuscript are fully available without restriction?	
**Describe where the data may be found in full sentences. If you are copying our sample text, replace any instances of XXX with the appropriate details.**<br><br>• If the data are **held or will be held in a public repository**, include URLs, accession numbers or DOIs. If this information will only be available after acceptance, indicate this by ticking the box below. For example: *All XXX files are available from the XXX database (accession number(s) XXX, XXX.*).<br>• If the data are all contained **within the manuscript and/or Supporting Information files**, enter the following: *All relevant data are within the manuscript and its Supporting Information files.*<br>• If neither of these applies but you are able to provide **details of access elsewhere**, with or without limitations, please do so. For example:	All relevant data are within the manuscript and its Supporting Information files.

Powered by Editorial Manager® and ProduXion Manager® from Aries Systems Corporation

Data cannot be shared publicly because of [XXX]. Data are available from the XXX Institutional Data Access / Ethics Committee (contact via XXX) for researchers who meet the criteria for access to confidential data.<br><br>*The data underlying the results presented in the study are available from (include the name of the third party and contact information or URL).*<br>• This text is appropriate if the data are owned by a third party and authors do not have permission to share the data.<br><br>* typeset	
Additional data availability information:	

Powered by Editorial Manager® and ProduXion Manager® from Aries Systems Corporation

Manuscript
Click here to access/download;Manuscript;Sami_manuscript.docx

Rehabilitation outcomes following tail-fluke amputation in an Indo-Pacific bottlenose dolphin: a welfare-centered approach

Suguru Higa[1], Sayaka Takahashi[1], Eri Nakashima[1], Yui Kurosu[1], Haruka Ikeshima[1], Ryota Yagi[1], Nihiro Adachi[1], Keiichi Ueda[1], Hirobumi Umeyama[2], Hitoshi Yamamoto[2], Yukinori Nakakita[2], Kazuma Tochigi[2], Taihei Kagawa[2], Shin-ichiro Oka[1*]

[1] Aquarium Management Center, Okinawa Churashima Foundation, Motobu, Okinawa, Japan

[2] Bridgestone Corporation, Chuo-ku, Tokyo, Japan

*Corresponding author

E-mail: sh-oka@okichura.jp (SO)

Abstract

Tail fluke loss in cetaceans compromises locomotion and impairs social functioning, posing serious welfare challenges. Rehabilitation strategies that address both physical performance and behavioral reintegration are essential for improving quality of life in affected individuals. This study evaluated the outcomes of a structured, two-phase rehabilitation program applied to Sami, an adult Indo-Pacific bottlenose dolphin (*Tursiops aduncus*) that underwent tail-fluke amputation, with an emphasis on locomotor function, behavioral adaptation, and long-term welfare. The rehabilitation protocol comprised two phases. Phase 1 focused on restoring vertical tail-beat locomotion through a combination of range-of-motion exercises and the use of a custom-designed prosthetic tail fluke. Swimming performance was quantitatively assessed using biologging devices under three conditions: without tail flukes, with the prosthetic tail, and in healthy conspecifics. Phase 2 introduced structured cohabitation with familiar individuals to promote social reintegration. Behavioral data were collected before and after rehabilitation to evaluate affiliative engagement and activity patterns. While maximum swim speed remained lower than in healthy individuals, propulsion per stroke significantly improved with prosthetic use. Notably, the subject dolphin retained species-typical vertical tail-beat motion even after prosthesis discontinuation, indicating

motor pattern adaptation. Post-rehabilitation, affiliative behavior increased to 17% of total observed activity—more than twice that of a healthy control—while resting behavior markedly declined. No aggression or abnormal behaviors were observed. This study demonstrates that a welfare-centered, multi-phase rehabilitation framework can effectively promote both functional recovery and social reengagement in dolphins with severe caudal injuries. The long-term retention of adaptive locomotor and social behaviors highlights the potential of integrative approaches to enhance the quality of life in physically compromised cetaceans.

Introduction

In recent years, the concept of animal welfare has gained significant attention in marine biology and captive animal care. Ensuring the physical and psychological well-being of animals under human care is now a central priority [1–3]. The current framework of animal welfare evaluation increasingly incorporates the multidimensional perspective described in the Five Domains model [4]. Among marine mammals, dolphins are known for their advanced intelligence, strong social bonds, and specific physical needs critical for maintaining their quality of life (QOL) [2,5,6]. However, severe injuries such as the loss of tail flukes pose significant challenges to their mobility, social integration, and

overall welfare, underscoring the need for innovative rehabilitation strategies. While anecdotal reports have documented the application of prosthetic devices in dolphins—such as in cases at the Okinawa Churaumi Aquarium and Clearwater Marine Aquarium—these interventions have not been systematically evaluated [2,7]. In particular, long-term outcomes related to psychological well-being, social reintegration, and behavioral adaptability remain largely unexplored.

Globally, recent reviews have emphasized that marine mammal rehabilitation efforts still lack standardized clinical frameworks and consistent reporting of welfare outcomes [8]. These studies also highlight the need for welfare-centered, evidence-based rehabilitation protocols and transparent post-treatment evaluations. Building on this context, the present study addresses these challenges by providing a structured, welfare-focused rehabilitation case involving an Indo-Pacific bottlenose dolphin (*Tursiops aduncus*) that underwent tail-fluke amputation followed by prosthetic adaptation.

To our knowledge, this represents the first systematic assessment of such an approach in this species. The research aims to evaluate the impact of a prosthetic tail on restoring swimming ability, enhancing social reintegration, and improving overall welfare. By combining detailed behavioral observations with objective assessments of psychological and social outcomes, this study advances the understanding of holistic welfare approaches

in marine mammal rehabilitation.

This work also holds broader significance. It demonstrates how integrating welfare-centered practices with technological innovation can address the complex needs of injured marine animals. Additionally, it contributes to the development of standardized methods for assessing animal welfare, providing a model for facilities worldwide. By bridging gaps in knowledge and practice, this study not only improves the quality of life for dolphins like the one in this case but also paves the way for more effective and compassionate care for marine mammals globally.

Although this study focuses on a single individual, it is designed not as an anecdotal case report but as a hypothesis-driven, systematically structured investigation incorporating quantitative and behavioral assessments. Given the rarity and ethical limitations associated with studying severe caudal injuries in cetaceans, such opportunities for longitudinal evaluation under controlled conditions are extremely limited. Therefore, this study serves as a scientifically grounded pilot for evaluating rehabilitation strategies that are potentially generalizable to other individuals or species under professional care. Our goal is to contribute a reproducible framework that can inform future multi-individual studies and enhance rehabilitation practices across institutions.

Materials and Methods

Ethical statement

This study was conducted in accordance with the Ethical Guidelines for the Conduct of Research on Animals by Zoos and Aquariums issued by the World Association of Zoos and Aquariums (WAZA) and the Code of Ethics established by the Japanese Association of Zoos and Aquariums (JAZA). It also adhered to the Animal Care Guidelines of the Okinawa Churashima Foundation, which oversees the Okinawa Churaumi Aquarium. Additionally, the study was approved by the Animal Experiment Committee established by the Okinawa Churashima Foundation (Permission Number: AT22003). These guidelines and committee oversight emphasize the importance of a thorough understanding and careful management of animal welfare. The acts involved in this study did not negatively impact the welfare of the dolphins involved.

Subject Dolphin

The subject of this study was Sami, a captive-born adult female Indo-Pacific bottlenose dolphin (*Tursiops aduncus*) housed at the Okinawa Churaumi Aquarium. In February 2021, she underwent surgical amputation of the tail flukes due to trauma-induced necrosis.

The rehabilitation process enabled a structured evaluation of prosthetic-assisted recovery in cetaceans with severe caudal injury, focusing on swimming function, social reintegration, and overall welfare.

In September 2020, the subject dolphin sustained a significant blunt trauma to her tail flukes after colliding with the pool wall. The resulting injury developed into a complex infection, leading to progressive necrosis of the affected tissue. On February 2021, post-injury, the tail flukes were surgically amputated, leaving only the central portion intact. The impact also caused a rupture of the ventral tendon on the right side of the caudal vertebrae, resulting in an asymmetrical deformation of the tail. Specifically, the distal portion of the vertebrae became twisted upward and to the right (Fig 1).

Fig 1. Tail flukes amputation and prosthetic application in the Indo-Pacific bottlenose dolphin. (A) Lateral view of the subject dolphin following surgical removal of the tail flukes due to trauma-induced necrosis. (B) Close-up of the residual tail stump showing asymmetrical deformation. (C) Custom-designed prosthetic tail flukes developed for attachment to the caudal peduncle. (D) The dolphin swimming with the prosthetic tail flukes attached, exhibiting vertical tail-beat motion.

Following the completion of the surgical amputation in June 2021, the subject dolphin initially attempted to swim using a lateral (side-to-side) tail motion. However, this failed to generate sufficient propulsion, and her voluntary swimming activity gradually declined. At the same time, her engagement in social interactions with other dolphins also decreased markedly. This state persisted for approximately one month. At the time of the incident, the subject dolphin measured 237 cm in length and weighed 143 kg.

Rehabilitation 1: Restoration of Swimming Ability Using a Prosthetic Tail flukes

In line with the case of Fuji, a Pacific bottlenose dolphin at our aquarium whose quality of life (QOL) was significantly improved through the use of prosthetic tail flukes [2], this study prioritized the restoration of swimming ability through the application of a prosthetic device. The subject dolphin had an asymmetrical distal end of the tail stump, requiring a secure attachment system capable of withstanding the forces generated during propulsion. A socket-type attachment was designed to encase the entire caudal peduncle, onto which rubber prosthetic tail flukes—shaped to resemble natural flukes—were mounted (Fig 1, S1 Video).

Following the loss of her tail flukes, the subject dolphin developed a compensatory

swimming pattern characterized by pronounced lateral body undulation (S2 Video). While this strategy may have enabled limited locomotion, it imposed abnormal strain on muscle groups and tendons not typically engaged during normal cetacean swimming. To mitigate the risk of injury and to support functional recovery, rehabilitation efforts focused on reestablishing a species-typical stroke pattern involving vertical tail movements, which are essential for efficient propulsion and overall biomechanical balance.

To address this, a rehabilitation program was implemented between July 2021 and May 2022 to increase vertical tail flexibility and restore an appropriate stroke axis. During these sessions, the pool was drained and the dolphin's body was gently restrained. A single trainer manually guided the distal tail region through a full vertical range of motion—up and down—until natural resistance was encountered. Each session lasted approximately 20 minutes and was conducted one to five times per week, for a total of 30 sessions (S3 Video). No aversive or avoidance behaviors were observed during this phase.

Following this stretching rehabilitation, habituation training to the prosthetic device was carried out between May and December 2022. The prosthetic tail was attached, and the subject dolphin was allowed to swim freely in her exhibit pool for 5 to 15 minutes per session. This training was conducted 1 to 5 times per week for a total of 43 sessions. The

goal of this phase was to promote physical adaptation and establish a positive behavioral association with the prosthesis.

Evaluation of Swimming Efficiency Using Prosthetic Tail flukes

To evaluate the effect of a prosthetic tail on swimming performance, we compared three conditions: (1) a dolphin without tail flukes (the subject dolphin), (2) the same dolphin with prosthetic tail flukes, and (3) a healthy individual of the same species. In all trials, the dolphins were encouraged to swim at maximum effort along the perimeter of the pool. Swimming efficiency was assessed using data recorded by animal-borne data loggers.

The loggers used were OR1400-3MPD3GT units manufactured by Little Leonardo Corporation. Two loggers were attached to each dolphin using suction cups—one placed just below the dorsal fin to record swim speed, and the other near the tail region to measure stroke frequency based on the periodicity of acceleration along the z-axis. Data loggers recorded swim speed, stroke frequency, and tri-axial acceleration (x, y, and z axes). In the present analysis, the periodicity of acceleration along the longitudinal (z) axis was used to calculate stroke frequency, while swim speed was obtained from the synchronized data.

For the subject dolphin, nine trials were conducted without the prosthetic tail flukes and eight trials with it. In addition, nine trials were conducted in total with two healthy Indo-Pacific bottlenose dolphins. For each trial, we extracted one complete tail stroke corresponding to the moment of maximum swim speed for analysis.

Swimming efficiency was quantified using the relative propulsion distance per stroke (RPDS), a dimensionless index defined as:

$$\mathrm{RPDS} = \frac{\text{Swim speed (m s}^{-1}\text{)}}{\text{Body length (m)} \times \text{Stroke frequency (storke s}^{-1}\text{)}}$$

This index represents the relative distance traveled per stroke, normalized by body length and stroke frequency. Bainbridge [9] demonstrated a strong relationship between swim speed, body length, and tail beat frequency, supporting the validity of these parameters. The RPDS was therefore considered a particularly suitable indicator for evaluating and comparing the swimming efficiency of a dolphin without tail flukes, one equipped with prosthetic tail flukes, and healthy conspecifics.

However, the evaluation of swimming efficiency using the prosthetic tail was discontinued in January 2023 to prevent the progression of a localized infection observed at the distal end of the tail stump. This decision was made as a precautionary medical

measure, and the prosthetic tail has not been used since, to allow for proper treatment and healing.

Statistical comparisons of locomotor parameters among the rehabilitation phases were conducted using one-way analysis of variance (ANOVA) followed by Tukey's honest significant difference (HSD) post hoc test. All analyses were performed in Python (version 3.10) using standard scientific libraries.

Rehabilitation 2: Restoration of Social Behavior

Social relationships are known to play a critical role in the welfare of Indo-Pacific bottlenose dolphins [5,6]. Following the surgical removal of the tail flukes, the subject dolphin was housed alone throughout Rehabilitation Phase 1, which focused on restoring swimming ability. During this period, a short-term cohabitation trial was conducted with a familiar conspecific that had previously lived with the subject dolphin. The aim of this trial was to promote the reacquisition of natural social behaviors. However, the attempt was discontinued due to the emergence of aggressive behaviors, such as chasing and physical contact directed toward the subject dolphin, as she was unable to synchronize her movements with the healthy individual.

As Rehabilitation Phase 1 progressed, the subject dolphin began to exhibit consistent,

voluntary swimming characterized by stable vertical tail movements, indicating a recovery of motivation. Based on this improvement, a second short-term cohabitation trial was implemented. This time, no aggressive or avoidance behaviors (e.g., chasing or withdrawal) were observed. In light of this outcome, a gradual reintroduction to social interactions was initiated as Rehabilitation Phase 2, with the goal of restoring social engagement.

Rehabilitation Phase 2 was conducted over a period of 53 days between September and October 2024. The subject dolphin was housed in a large outdoor pool (maximum diameter: 25 m; depth: 6 m; water volume: 1,750 m^3) together with a diverse group of conspecifics and related species, including *Tursiops adu*ncus, *Tursiops truncat*us, Pseudorca crassidens, and *Steno bredanensis*—all female individuals with whom she had previously established affiliative relationships. To promote the restoration of sociality through natural interactions, no special treatment was given to the subject dolphin during the cohabitation period, and human intervention in inter-animal dynamics was deliberately avoided. The number of cohabiting individuals (excluding the subject dolphin) fluctuated throughout the rehabilitation period due to management requirements: four animals were present for 26 days, five for 22 days, three for 3 days, and six for 2 days. Qualitative behavioral observations were conducted five times daily, generally

aligned with the five scheduled feeding sessions. These observations focused on spontaneous interactions, behavioral changes, and social dynamics among dolphins and served as a basis for evaluating the progression of her social reintegration in a naturalistic setting.

To quantitatively assess behavioral changes before and after Rehabilitation Phase 2, focal observations were conducted on the subject dolphin over two distinct periods: four days between November 2021 and January 2022 (pre-rehabilitation), and three days between October and November 2024 (post-rehabilitation). During the pre-rehabilitation period, the subject dolphin was housed with a female rough-toothed dolphin (*Steno bredanensis*) and bottlenose dolphins (*Tursiops truncatus*), while in the post-rehabilitation period, she was cohabiting with female bottlenose dolphins and false killer whales (*Pseudorca crassidens*). All cohabiting animals had previously exhibited affiliative relationships with the subject dolphin prior to her injury. In both periods, cohabitation took place in an outdoor tank measuring 14 meters in length, 3 meters in depth, and with a water volume of 400 m^3.

The focal animal's behavioral state was recorded every 5 minutes over a 1-hour period using scan sampling. Observations were consistently conducted from 12:30 to 13:30, a non-feeding period, to avoid the influence of feeding motivation. Because the behavioral

state was recorded objectively at fixed 5-minute intervals using scan sampling, observer bias and recording errors were minimized. All sessions were conducted while the dolphin was not wearing prosthetic tail flukes.

To evaluate the dolphin's psychological and social state, six behavioral categories were used, based on the definitions provided by Miller et al.[6]: resting, affiliative behavior, aggressive behavior, repetitive behavior, high-energy behavior, and others (e.g., routine swimming, object play, exploratory behavior). As a behavioral control, the same behavioral observations were also conducted on a healthy adult female Indo-Pacific bottlenose dolphin over seven days between October 2024 and January 2025. This individual had been under human care at the Okinawa Churaumi Aquarium since 1975 and was the biological mother of the subject dolphin. While her exact age is unknown, her long-term residency and environmental familiarity were considered appropriate for use as a behavioral reference in this study.

Result

Rehabilitation Phase 1

Rehabilitation Phase 1, which focused on expanding the vertical range of tail motion and correcting the stroke axis from horizontal to vertical, required approximately 11 months

to complete. As a result of this intervention, the subject dolphin regained the ability to swim with a consistent vertical tail beat. Habituation to the prosthetic tail flukes also progressed smoothly. Within approximately 3 months from the start of this phase, the subject dolphin demonstrated stable and coordinated swimming while wearing the prosthetic (S4 Video). Notably, even when not wearing the prosthetic tail flukes, the subject dolphin no longer exhibited the snake-like lateral body undulation that was previously observed immediately following tail loss. This behavioral change suggests that the vertical tail stroke pattern was retained even in the absence of the prosthetic device, indicating the effectiveness of the rehabilitation in promoting lasting motor adaptation.

Swimming Efficiency Using Prosthetic Tail flukes

Fig 2 illustrates the results of high-speed swimming trials under three conditions: without tail flukes (No Fin), with prosthetic tail flukes, and in a healthy control individual. The maximum swimming speed was significantly lower in both the no fin and prosthetic conditions compared to the control group ($p < 0.001$), with no significant difference between the no fin and Prosthetic groups.

Fig 2. Boxplots of maximum swim speed (A), stroke frequency (B), and relative

propulsive distance per stroke (RPDS) (C) measured by accelerometer under three conditions: No Fin, Prosthetic Tail, and Control (a healthy individual with intact tail flukes). Asterisks indicate statistically significant differences between groups based on Tukey's HSD test ($p < 0.05$). Boxes represent the interquartile range (IQR), horizontal lines inside boxes indicate medians, and whiskers extend to 1.5×IQR. Outliers are plotted as individual points.

Stroke frequency, measured as the number of tail beats per second, did not differ significantly between the no fin and control groups ($p = 0.53$), but was significantly reduced in the prosthetic condition compared to both no fin and control ($p < 0.001$).

Relative propulsion distance per stroke (RPDS), which quantifies body-length-normalized distance traveled per tail stroke, differed significantly among all three groups ($p < 0.001$). The no fin condition showed the lowest RPDS values, while the prosthetic condition was significantly higher and closer to Control levels. In some trials, RPDS values in the prosthetic condition were comparable to or even exceeded those observed in healthy individual.

These results indicate that while the prosthetic tail did not contribute substantially to increasing swim speed—likely due to increased drag and reduced flexibility—it partially

restored propulsion efficiency lost due to tail loss. Notably, even though stroke frequency was reduced in the prosthetic condition, the distance traveled per stroke was improved, supporting the effectiveness of the prosthetic tail as a functional assistive device for locomotion.

Since January 2023, the use of the prosthetic tail has been discontinued for medical reasons. Nevertheless, until June 2025, when the individual died due to unrelated chronic conditions, primarily pulmonary in nature., the subject dolphin consistently maintained the vertical tail-beat swimming pattern acquired through rehabilitation, suggesting that the motor adaptation had been retained in the long-term.

Rehabilitation Phase 2

During Rehabilitation Phase 2, which aimed to restore social behaviors through active cohabitation, affiliative interactions began to emerge shortly after the initiation of the program. Behaviors such as synchronous swimming and close-contact positioning—completely absent prior to rehabilitation—were consistently observed. In addition, instances of drafting behavior—defined as one individual swimming in the hydrodynamic wake of another—were documented during cohabitation (S5 Video). As this behavior entails coordinated movement and sustained proximity, it is considered a form of

affiliative interaction indicative of social cohesion. Quantitative behavioral analysis (Fig 3) revealed that prior to rehabilitation, the subject dolphin spent most of the observation time resting motionlessly at the surface, with no affiliative or social interactions recorded. In contrast, near the end of Rehabilitation Phase 2, the proportion of resting behavior had markedly decreased, while the "Others" category nearly doubled. Notably, affiliative behaviors accounted for approximately 17% of total observed behaviors. Within the "Others" category, the majority consisted of solitary swimming, with a smaller portion comprising exploratory and solitary play behaviors.

Fig 3 Behavioral composition of a rehabilitated Indo-Pacific bottlenose dolphin before and after rehabilitation, compared with a healthy control. Pie charts represent the proportion of six behavioral categories—resting, affiliative, high-energy, and others—observed during focal sampling sessions. “Others” includes routine swimming, solitary play, and exploratory behaviors.

Compared to a healthy adult *Tursiops aduncus* observed under similar conditions, the subject dolphin demonstrated more than double the frequency of affiliative behavior. No aggressive or repetitive behaviors were recorded throughout the observation period,

suggesting that the subject dolphin had re-established social engagement in a stable and positive manner.

Remarkably, these affiliative behaviors—including synchronous swimming and close-contact positioning—were observed even during the final stage of her life, while she was affected by a pulmonary condition, continuing up to the day before her death.

Discussion

Ethical Considerations in Rehabilitation

Ethical considerations were integral to decision-making throughout the rehabilitation process. The duration of prosthesis use was determined through continuous medical assessment and welfare monitoring, and the device was ultimately discontinued in accordance with the precautionary principle to prevent further infection at the stump site. Similarly, the timing of social reintroduction was guided by the dolphin's behavioral stability, health status, and stress indicators to ensure optimal welfare conditions. These decisions were made collaboratively by veterinarians and animal keepers, reflecting a welfare-centered and precautionary approach to rehabilitation.

Evaluation of Physical Recovery and Social Behavior

The rehabilitation program implemented for the subject dolphin resulted in significant improvements in both her physical performance and social engagement. In terms of swimming ability, the structured training allowed her to recover a vertical tail-beat locomotion, which had been lost following the injury. Although swimming speed in both the No Fin and Prosthetic conditions remained significantly lower than in healthy dolphins (Fig 2), the use of the prosthetic tail partially restored propulsion efficiency, as evidenced by a significantly higher RPDS compared to the No Fin condition. These findings indicate that while the prosthetic tail did not substantially improve speed, likely due to increased drag and reduced flexibility, it served as a functional assistive device that improved swimming efficiency per stroke. Notably, the subject dolphin continued to exhibit a stable vertical tail-beat pattern even after the prosthesis had been discontinued due to medical considerations, suggesting lasting motor adaptation.

In the domain of social behavior, progress was even more striking. At the end of Rehabilitation Phase 2, affiliative behaviors—such as synchronous swimming and close-contact positioning—accounted for approximately 17% of total observed behavior (Fig 3). Prior to rehabilitation, such interactions were entirely absent, and the subject dolphin spent nearly all her time resting motionlessly at the surface. In contrast, post-rehabilitation

observations showed a substantial reduction in resting and an increase in active behaviors, including solitary swimming, exploratory movement, and solitary play. Within the "Others" category, the majority consisted of solitary swimming, with a smaller portion comprising exploratory and solitary play behaviors.

Moreover, her social activity time fell within the range observed in wild dolphins, which spend approximately 4–31% of their time engaged in social behaviors [6]. Compared with a healthy adult *Tursiops aduncus* observed under similar conditions, the subject dolphin demonstrated more than double the frequency of affiliative behavior. Her successful reintegration into the social group underscores the rehabilitation program's effectiveness in addressing both physical and social challenges, and it contributed significantly to her overall recovery and quality of life (QOL). Notably, both the vertically undulating swimming pattern and affiliative behaviors were retained until the final stage of her life, even after the progression of a fatal pulmonary condition. The long-term retention of vertical locomotion and affiliative behaviors indicates the potential for sustained adaptation following targeted rehabilitation interventions.

Psychological Changes and Resilience

Beyond these physical and behavioral outcomes, the rehabilitation process appeared to

foster notable psychological changes. The repeated success in adapting to the prosthetic tail flukes, followed by the maintenance of learned motor patterns even after its discontinuation, may be indicative of enhanced self-efficacy—the belief in one's ability to perform essential tasks [10]. This concept, originally developed in human psychology, has gained increasing relevance in the context of animal rehabilitation, particularly for cognitively advanced species such as dolphins [11].

Another critical factor in her recovery was the successful generalization of learned behaviors and the restoration of a species-typical movement pattern. Skills acquired during prosthetic-assisted swimming—such as vertical tail-beat motion and coordinated body control—were effectively transferred to situations where the prosthesis was no longer used. This continuity suggests the development of robust motor learning and supports Shepard's theory of generalization [12], whereby behaviors learned in one condition can be applied to analogous situations through shared psychological processes. Importantly, the regained vertical tail movement may have played a key role not only in sustaining physical mobility but also in facilitating social reintegration. It is plausible that movement patterns consistent with species-typical norms contribute to social recognition and acceptance within animal groups. In some mammalian species, disruptions to such behavioral cues have been linked to social rejection or exclusion. For instance,

chimpanzees that exhibit aberrant or socially inappropriate behavior are known to be actively shunned by group members [13], and African elephants that experience early-life social disruption have shown non-normative behavior and difficulties in reintegrating into social groups due to deficits in learned social and motor patterns [14]. Furthermore, abnormal locomotor patterns—especially those that deviate from species-typical mechanics—may trigger avoidance or aggression from peers [15] By regaining vertical tail-beat locomotion, the subject dolphin may have reduced the perceptual incongruence that could otherwise have led to negative social responses, ultimately promoting social acceptance and the emergence of affiliative behaviors during cohabitation.

The Role of Positive Reinforcement

Throughout both rehabilitation phases, positive reinforcement played a central role. Reward-based training not only facilitated prosthetic adaptation but also reinforced a positive association with swimming and physical engagement. The continuation of voluntary swimming and exploration after the discontinuation of the prosthesis further suggests that intrinsic motivation had been established. Parallels can be found in human and non-human primate rehabilitation, where structured positive reinforcement has been shown to enhance motivation, reduce stress, and improve adaptability [16,17]. These

findings underscore the cross-species relevance of reinforcement-based methodologies in promoting behavioral resilience and psychological well-being.

Implications for Animal Welfare and Rehabilitation

These findings emphasize the importance of a holistic approach to rehabilitation that addresses both physical and psychological domains. Reintegration into a social group was a critical component of recovery, as social affiliation and culturally transmitted behaviors are vital to the well-being of cetaceans [18]. In highly social species like the Indo-Pacific bottlenose dolphin, providing opportunities for meaningful social engagement is essential for long-term welfare. The observed resilience and adaptability further highlight the role of cognitive processing in animal welfare, as discussed by Duncan and Petherick [15]. Structured rehabilitation programs that incorporate opportunities for learning, problem-solving, and autonomous interaction can enhance not only physical recovery but also psychological health and behavioral flexibility.

Although prosthetic applications in marine mammals other than dolphins have not been reported, similar attempts have been made in sea turtles that have lost their flippers. These studies, however, have largely focused on the engineering and hydrodynamic aspects of prosthetic design rather than on behavioral or welfare outcome [19,20]. In contrast, the

present study demonstrates how prosthetic technology can be integrated into a welfare-centered rehabilitation program that simultaneously addresses locomotor function, social behavior, and psychological recovery.

The observed outcomes underscore the value of structured, welfare-centered interventions in promoting recovery among cetaceans with severe injuries. The use of multidimensional metrics—including activity patterns, social behavior, and adaptive functioning—may support more standardized and species-appropriate assessments of rehabilitation success. Such frameworks could improve not only the design of future interventions but also transparency and accountability in animal welfare practices [21].

Although the subject dolphin showed sustained behavioral stability and social engagement following rehabilitation, ensuring long-term welfare remains crucial when individuals have undergone severe physical trauma. Continuous assessment of physical condition, social relationships, and behavioral diversity can help detect subtle signs of discomfort, social isolation, or chronic stress. Welfare frameworks such as the Five Domains model [4] highlight the need for multidimensional evaluation and management responsive to each animal's mental state. Moreover, recent reviews of marine mammal rehabilitation emphasize the paucity of robust long-term post-release and welfare data [8], reinforcing the imperative for ongoing monitoring. Adaptive management—guided by

systematic welfare data—allows caretakers to modify group composition, enrichment programs, or medical interventions in response to changing welfare needs. Establishing longitudinal welfare datasets across facilities would further enhance our understanding of recovery trajectories and support evidence-based refinement of rehabilitation practices for marine mammals.

Practical Guidelines for Dolphin Rehabilitation

Recent global reviews have highlighted the lack of standardized clinical frameworks and quantitative welfare assessments in marine mammal rehabilitation programs [8]. This study presents a structured, welfare-centered protocol that directly addresses this gap, providing a practical example of how standardization can be implemented in real-world settings. The experience from this case offers practical insights for facilities managing dolphins with severe caudal injuries.

A two-phase rehabilitation framework—comprising physical and social recovery—proved effective in guiding clinical decisions and monitoring progress.

During Phase 1 (physical rehabilitation), wound healing and muscle recovery should be prioritized, and the timing of prosthesis introduction should be determined cautiously, considering infection risk and skin tolerance.

During Phase 2 (social rehabilitation), gradual reintroduction to conspecifics and careful behavioral monitoring are essential to ensure social stability and psychological well-being.

Establishing objective behavioral and locomotor indicators to assess progress at each phase would further strengthen the reproducibility of such approaches. These recommendations could serve as a practical foundation for developing future standardized clinical protocols in marine mammal rehabilitation.

Implementation of such rehabilitation programs requires coordinated collaboration among veterinarians, animal trainers, and engineers to ensure safety and welfare at each stage. Staff should receive specialized training in prosthetic handling, behavioral monitoring, and welfare assessment to minimize stress during interventions. Facilities must be equipped with adaptable pools that allow controlled water depth for physical therapy, as well as access to materials and fabrication expertise for custom prosthetic design. Although initial development costs—such as prosthetic fabrication and data-logging equipment—can be considerable, these are often offset by the long-term welfare benefits and improved management efficiency achieved through structured rehabilitation. Sharing technical resources and training programs among institutions

would help reduce costs and promote wider adoption of welfare-centered rehabilitation frameworks.

Conclusions

This study evaluated the outcomes of a structured rehabilitation program for a tail-fluke-amputated Indo-Pacific bottlenose dolphin through a structured, multi-phase program aimed at restoring swimming ability, social integration, and psychological well-being. We hypothesized that a welfare-centered intervention combining prosthetic-assisted locomotor training and socially enriching environments could lead to long-term functional and behavioral recovery. Our findings support this hypothesis.

The use of custom-made prosthetic tail flukes significantly improved propulsion per stroke (RPDS), even though absolute swim speed remained suboptimal due to mechanical constraints. Notably, vertical tail-beat locomotion was retained even after prosthesis discontinuation, indicating durable motor adaptation. Socially, affiliative behavior increased to levels surpassing those observed in healthy conspecifics, with no aggression or abnormal behaviors recorded, suggesting a complete reintegration into the social group. These improvements were sustained until the final stage of life, highlighting the lasting impact of rehabilitation.

This study contributes new insights to the field of marine mammal welfare by (1) providing quantitative evidence of long-term biomechanical and social adaptation following tail loss, (2) demonstrating the value of prosthetic training beyond physical function, including psychological resilience and enhanced self-efficacy, and (3) proposing a framework that integrates physical, social, and psychological metrics for evaluating rehabilitation success.

Limitations of this study include its single-subject design and the inability to assess long-term prosthetic use due to medical decisions aimed at preventing the progression of infection. While these constraints are inherent to case-based studies, they also highlight the need for broader, collaborative efforts to validate and extend the present findings. To overcome the limitations of single-case studies, future research should involve multi-institutional collaborations that allow comparative and longitudinal assessments of prosthetic adaptation and social reintegration. Establishing shared behavioral metrics and rehabilitation records across facilities would enable the identification of individual variability and key factors for success, contributing to a more generalizable welfare assessment framework. Such collaborative efforts will strengthen the scientific basis of marine mammal rehabilitation and promote higher international standards of welfare practice. It is also worth speculating that improved motor patterns may have facilitated

social acceptance by reducing visual incongruence—a hypothesis that warrants further ethological investigation.

Taken together, this study underscores the feasibility and necessity of holistic rehabilitation approaches for cetaceans with severe physical trauma. By addressing mobility, sociality, and emotional health in tandem, such programs can meaningfully improve quality of life and serve as a model for welfare-driven innovation in marine animal care.

Acknowledgement

We thank the staff at the Okinawa Churaumi Aquarium for animal training, handling, and medical treatment. Koji Tokutake and Dr. Isao Kawazu, former managers of the marine mammal department, provided encouragement and support throughout this project. We also thank Bridgestone Corporation for their generous financial and resource support in the development of the prosthetic tail. We are also grateful to the staff of the Wildlife Research Center of Kyoto University for kindly providing the data loggers used to measure swimming performance. We also express our sincere respect to Sami, the Indo-Pacific Bottlenose Dolphin who participated in this study. Although she passed away due to a lung disease during the manuscript preparation, her contributions to our

understanding of physical and social recovery in marine mammals remain invaluable. Artificial intelligence assistance (ChatGPT, OpenAI) was used solely for language polishing and improving the clarity of the English text. All scientific content, data analysis, and conclusions were developed entirely by the authors.

References

1. Miller L, Mellen J, Greer T, Ii SK. The effects of education programmes on Atlantic bottlenose dolphin *(Tursiops truncatus)* behaviour. Anim welf. 2011;20: 159–172. doi:10.1017/S0962728600002645

2. Ueda K, Murakami M, Kato J, Miyahara H, Izumisawa Y. Intervention to improve the quality of life of a Bottlenose dolphin that developed necrosis on the tail flukes. J Phys Ther Sci. 2013;25: 1201–1207. doi:10.1589/jpts.25.1201

3. Kagan R, Carter S, Allard S. A. Universal animal welfare framework for zoos. Journal of Applied Animal Welfare Science. 2015;18: S1–S10. doi:10.1080/10888705.2015.1075830

4. Mellor DJ, Beausoleil NJ, Littlewood KE, McLean AN, McGreevy PD, Jones B, et al. The 2020 Five domains model: Including human–animal interactions in assessments of animal welfare. Animals. 2020;10: 1870. doi:10.3390/ani10101870

5. Lauderdale LK, Mellen JD, Walsh MT, Granger DA, Miller LJ. Towards understanding the welfare of cetaceans in accredited zoos and aquariums. Zaccaroni A, editor. PLoS ONE. 2021;16: e0255506. doi:10.1371/journal.pone.0255506

6. Miller LJ, Lauderdale LK, Mellen JD, Walsh MT, Granger DA. Relationships between animal management and habitat characteristics with two potential indicators of welfare for bottlenose dolphins under professional care. Li S, editor. PLoS ONE. 2021;16: e0252861. doi:10.1371/journal.pone.0252861

7. Clearwater Marine Aquarium. Winter the dolphin. n.d. Available: https://www.cmaquarium.org/animals/dolphins/winter/

8. Simeone CA, Rousselet E, Atkin C, De Trez M, Delemotte M, Johnson SP. A systematic review of global marine mammal rehabilitation and refloating, 2000–2023. Sustainability. 2024;16: 4589. doi:10.3390/su16114589

9. Bainbridge R. The speed of swimming of fish as related to size and to the frequency and amplitude of the tail beat. Journal of Experimental Biology. 1958;35: 109–133. doi:10.1242/jeb.35.1.109

10. Bandura A. Self-efficacy: Toward a unifying theory of behavioral change. Psychological Review. 1977;84: 191–215. doi:10.1037/0033-295X.84.2.191

11. Marino L. Convergence of complex cognitive abilities in cetaceans and primates.

Brain Behav Evol. 2002;59: 21–32. doi:10.1159/000063731

12. Shepard RN. Toward a universal law of generalization for psychological science. Science. 1987;237: 1317–1323. doi:10.1126/science.3629243

13. Goodall J. The chimpanzees of Gombe: patterns of behavior. Cambridge, Massachusetts London, England: The Belknap Press of Harvard University Press; 1986.

14. Shannon G, Slotow R, Durant SM, Sayialel KN, Poole J, Moss C, et al. Effects of social disruption in elephants persist decades after culling. Front Zool. 2013;10: 62. doi:10.1186/1742-9994-10-62

15. Duncan IJ, Petherick JC. The implications of cognitive processes for animal welfare. Journal of Animal Science. 1991;69: 5017–5022. doi:10.2527/1991.69125017x

16. Ryan RM, Deci EL. Self-determination theory and the facilitation of intrinsic motivation, social development, and well-being. American Psychologist. 2000;55: 68–78. doi:10.1037/0003-066X.55.1.68

17. Laule G, Whittaker M. Enhancing nonhuman primate care and welfare through the use of positive reinforcement training. Journal of Applied Animal Welfare Science. 2007;10: 31–38. doi:10.1080/10888700701277311

18. Rendell L, Whitehead H. Culture in whales and dolphins. Behav Brain Sci. 2001;24:

309–324. doi:10.1017/S0140525X0100396X

19. Van Der Geest N, Garcia L. Employing robotics for the biomechanical validation of a prosthetic flipper for sea turtles as a substitute for animal clinical trials. Biomechanics. 2023;3: 401–414. doi:10.3390/biomechanics3030033

20. Sun X, Kato N, Matsuda Y, Kanda K, Kosaka Y, Kamezaki N, et al. Three-dimensional hydrodynamic analysis of forelimb propulsion of sea turtle with prosthetic flippers. J Abmech. 2013;3: 36–44. doi:10.5226/jabmech.3.36

21. Scott EM, Nolan AM, Reid J, Wiseman-Orr ML. Can we really measure animal quality of life? Methodologies for measuring quality of life in people and other animals. Animal Welfare. 2007;16: 17–24.

Supporting information

S1_Video. This supplementary video illustrates the structure and attachment procedure of the artificial tail fluke used during Phase 2 of rehabilitation. The prosthesis consisted of a rubber tail fluke connected to a carbon fiber socket via a metal plate. The socket was divided into upper and lower sections, which were joined and secured with bolts. A silicone lining was inserted inside the socket to prevent abrasion of the caudal peduncle and to improve comfort and safety during attachment.

S2_Video. Video recording of the subject dolphin exhibiting pronounced lateral body undulation following tail fluke loss. Although a prosthetic tail is attached, the vertical tail-beat motion had not yet been adequately rehabilitated. As a result, the observed swimming behavior deviates from the typical pattern and imposes increased biomechanical strain on the axial body musculature.

S3_Video. Video footage depicting the rehabilitation procedure aimed at restoring vertical tail-beat motion. As described in the main text, the dolphin's body is gently restrained while a trainer manually guides the distal tail region through its full vertical range of motion to promote flexibility and reestablish proper stroke axis.

S4_Video. Video recording of the subject dolphin exhibiting stable and coordinated swimming while wearing the prosthetic tail flukes. The footage illustrates successful habituation to the device, achieved during Rehabilitation Phase 2.

S5_Video. Video footage of the subject dolphin engaged in affiliative social behaviors after Rehabilitation Phase 2. The clip shows consistent synchronous swimming and close-

contact positioning with conspecifics, as well as an instance of drafting behavior—defined as swimming in the hydrodynamic wake of another dolphin. These behaviors reflect the reestablishment of social cohesion and affiliative engagement following rehabilitation.

S6_Table. The raw data used to generate the figures and statistical results in the manuscript are provided as a supplementary file. This includes behavioral observations and swimming performance measurements necessary to replicate the analyses.

Figure 1

Click here to access/download;Figure;Fig1.eps

turnitin Página 46 de 96 - Entrega de escritura con IA Identificador de la entrega trn:oid:::14652:533329514

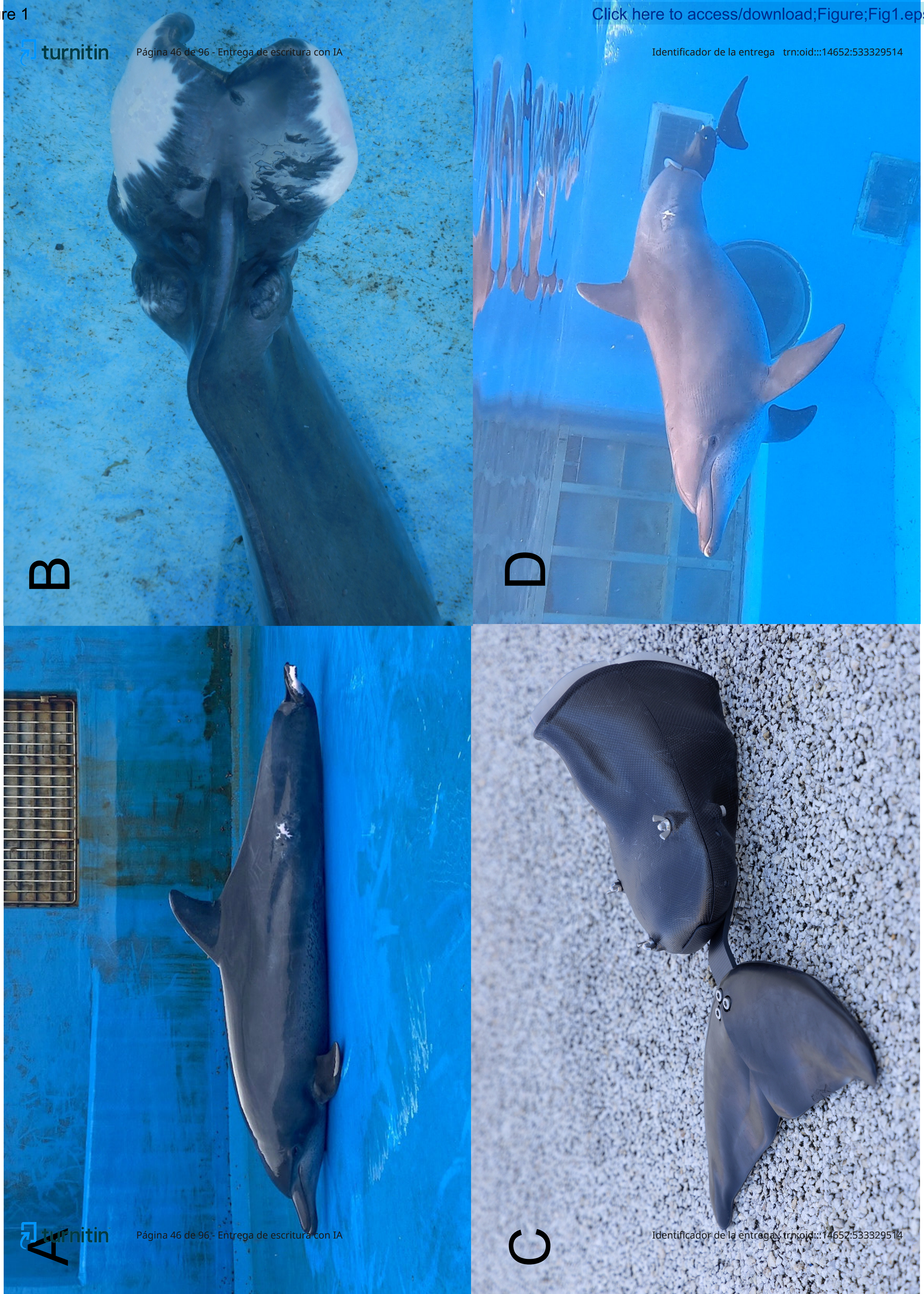

turnitin Página 46 de 96 - Entrega de escritura con IA Identificador de la entrega trn:oid:::14652:533329514

turnitin Página 47 de 96 - Entrega de escritura con IA Identificador de la entrega trn:oid:::14652:533329514

Figure 2

Click here to access/download;Figure;Fig2.eps

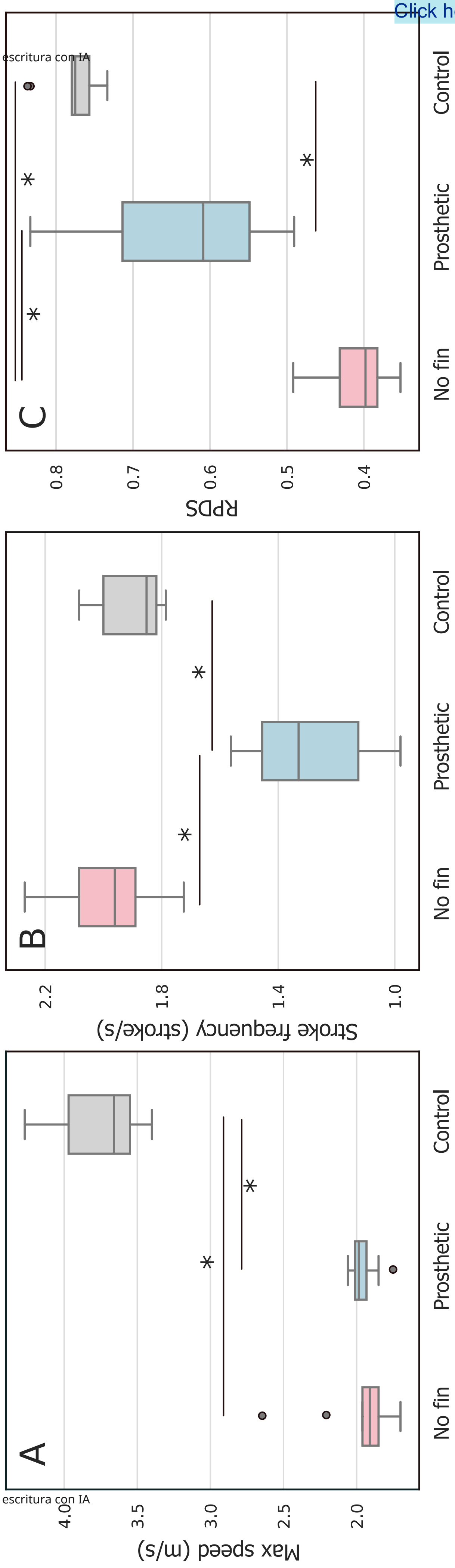

turnitin Página 47 de 96 - Entrega de escritura con IA Identificador de la entrega trn:oid:::14652:533329514

Figure 3

Click here to access/download;Figure;Fig3.eps

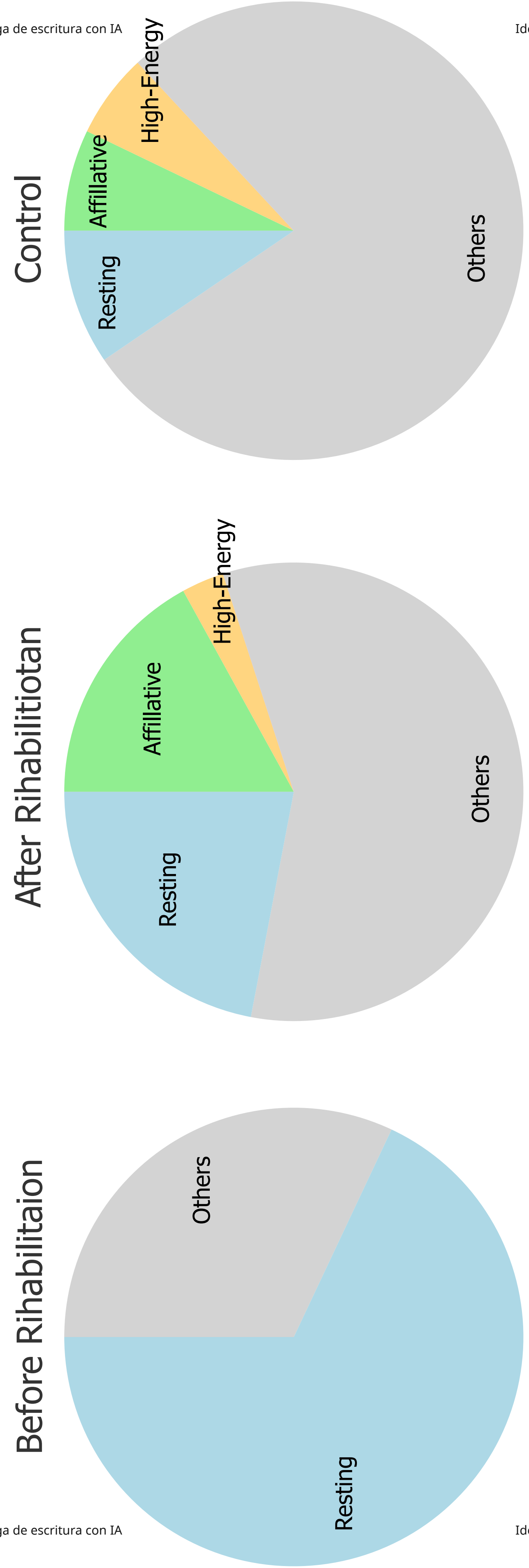

turnitin Página 49 de 96 - Entrega de escritura con IA Identificador de la entrega trn:oid:::14652:533329514

Click here to access/download
Supporting Information
S5_table.xlsx

turnitin Página 49 de 96 - Entrega de escritura con IA Identificador de la entrega trn:oid:::14652:533329514

S1_Video

Click here to access/download

Supporting Information

S1_video.mp4

Click here to access/download

Supporting Information

S2_video.mp4

S3_Video

Click here to access/download
Supporting Information
S3_video.mp4

S4_Video

Click here to access/download
Supporting Information
S4_video.mp4

S5_Video

Click here to access/download
Supporting Information
S5_video.mp4

S6_Table

Click here to access/download
Supporting Information
S6_table.xlsx

Rehabilitation outcomes following tail-fluke amputation in an Indo-Pacific bottlenose dolphin: a welfare-centered approach

Formatted: Font: (Default) Times New Roman, 18 pt

Formatted: Line spacing: Double

Suguru Higa[1], Sayaka Takahashi[1], Eri Nakashima[1], Yui Kurosu[1], Haruka Ikeshima[1], Ryota Yagi[1], Nihiro Adachi[1], Keiichi Ueda[1], Hirobumi Umeyama[2], Hitoshi Yamamoto[2], Yukinori Nakakita[2], Kazuma Tochigi[2], Taihei Kagawa[2], Shin-ichiro Oka[1*]

Formatted: Font: 12 pt

[1] Aquarium Management Center, Okinawa Churashima Foundation, Motobu, Okinawa, Japan

[2] Bridgestone Corporation, Chuo-ku, Tokyo, Japan

*Corresponding author

E-mail: sh-oka@okichura.jp (SO)

Abstract

Tail fluke loss in cetaceans compromises locomotion and impairs social functioning, posing serious welfare challenges. Rehabilitation strategies that address both physical performance and behavioral reintegration are essential for improving quality of life in affected individuals. This study evaluated the outcomes of a structured, two-phase rehabilitation program applied to Sami, an adult Indo-Pacific bottlenose dolphin (*Tursiops aduncus*) ~~following tail fluke~~that underwent tail-fluke amputation, with an emphasis on locomotor function, behavioral adaptation, and long-term welfare. The rehabilitation protocol comprised two phases. Phase 1 focused on restoring vertical tail-beat locomotion through a combination of range-of-motion exercises and the use of a custom-~~fitted~~ designed prosthetic tail fluke. Swimming performance was quantitatively assessed using biologging devices under three conditions: without tail flukes, with the prosthetic tail, and in healthy conspecifics. Phase 2 introduced structured cohabitation with familiar individuals to promote social reintegration. Behavioral data were collected before and after rehabilitation to evaluate affiliative engagement and activity patterns. While maximum swim speed remained lower than in healthy individuals, propulsion per stroke significantly improved with prosthetic use. Notably, the subject dolphin retained species-typical vertical tail-beat motion even after prosthesis discontinuation, indicating

Formatted: Font: 12 pt

Formatted: Indent: First line: 0.5 ch, Line spacing: Double

Formatted: Font: 12 pt

Formatted: Font: 12 pt

motor pattern adaptation. Post-rehabilitation, affiliative behavior increased to 17% of total observed activity—more than twice that of a healthy control—while resting behavior markedly declined. No aggression or abnormal behaviors were observed. This study demonstrates that a welfare-centered, multi-phase rehabilitation framework can effectively promote both functional recovery and social reengagement in dolphins with severe caudal injuries. The long-term retention of adaptive locomotor and social behaviors highlights the potential of integrative approaches to enhance the quality of life in physically compromised cetaceans.

Formatted: Line spacing: Double

Introduction

In recent years, the concept of animal welfare has gained significant attention in marine biology and captive animal care. Ensuring the physical and psychological well-being of animals under human care is now a central priority [1–3]. The current framework of animal welfare evaluation increasingly incorporates the multidimensional perspective described in the Five Domains model [4]. Among marine mammals, dolphins are known for their advanced intelligence, strong social bonds, and specific physical needs critical for maintaining their quality of life (QOL) [2,5,6]. However, severe injuries such as the loss of tail flukes pose significant challenges to their mobility, social integration, and

Formatted: Font: 12 pt

Formatted: Indent: First line: 0.5 ch, Line spacing: Double

Formatted: Font: (Default) Times New Roman, 12 pt

Formatted: Font: 12 pt

Formatted: Font: 12 pt

overall welfare, ~~raising~~ underscoring the need for innovative rehabilitation strategies.

While anecdotal reports have documented the application of prosthetic devices in dolphins—such as in cases at the Okinawa Churaumi Aquarium and Clearwater Marine Aquarium—these interventions have not been systematically evaluated [2,7]. In particular, long-term outcomes related to psychological well-being, social reintegration, and behavioral adaptability remain largely unexplored.

Globally, recent reviews have emphasized that marine mammal rehabilitation efforts still lack standardized clinical frameworks and consistent reporting of welfare outcomes [8]. These studies also highlight the need for welfare-centered, evidence-based rehabilitation protocols and transparent post-treatment evaluations. Building on this context, the present study addresses these challenges by providing a structured, welfare-focused rehabilitation case involving an Indo-Pacific bottlenose dolphin (*Tursiops aduncus*) that underwent tail-fluke amputation followed by prosthetic adaptation. ~~This highlights a critical need for holistic research that goes beyond physical recovery to assess the true effectiveness of such interventions.~~

~~This study evaluates a structured, welfare-centered rehabilitation protocol incorporating prosthetic-assisted locomotor training and behavioral reintegration, applied to an Indo-Pacific bottlenose dolphin (*Tursiops aduncus*).~~ To our knowledge, this represents the first

Formatted: Font: 12 pt

Formatted: Indent: First line: 0 ch, Line spacing: Double

Formatted: Font: 12 pt

Formatted: Font: Not Bold

Formatted: Font: (Default) Times New Roman, 12 pt, Not Bold

Formatted: Font: (Default) Times New Roman, 12 pt, Not Bold

Formatted: Font: (Default) Times New Roman, 12 pt

Formatted: Font: (Default) Times New Roman, 12 pt, Not Bold

Formatted: Font: (Default) Times New Roman, 12 pt, Not Bold

Formatted: Font: 12 pt

Formatted: Font: 12 pt

Formatted: Indent: First line: 0.5 ch, Line spacing: Double

systematic assessment of such an approach in this species. The research aims to evaluate the impact of a prosthetic tail on restoring swimming ability, enhancing social reintegration, and improving overall welfare. By combining detailed behavioral observations with objective assessments of psychological and social outcomes, this study advances the understanding of holistic welfare approaches in marine mammal rehabilitation.

This work also ~~research~~ holds broader significance. It demonstrates how integrating welfare-centered practices with technological innovation can address the complex needs of injured marine animals. Additionally, it contributes to the development of standardized methods for assessing animal welfare, providing a model for facilities worldwide. By bridging gaps in knowledge and practice, this study not only improves the quality of life for dolphins like the one in this case but also paves the way for more effective and compassionate care for marine mammals globally.

Formatted: Indent: First line: 0.5 ch, Line spacing: Double

Formatted: Font: 12 pt

Although this study focuses on a single individual, it is designed not as an anecdotal case report~~,~~ but as a hypothesis-driven, systematically structured investigation incorporating quantitative and behavioral assessments. Given the rarity and ethical limitations associated with studying severe caudal injuries in cetaceans, such opportunities for longitudinal evaluation under controlled conditions are extremely

limited. Therefore, this study serves as a scientifically grounded pilot for evaluating rehabilitation strategies that are potentially generalizable to other individuals or species ~~in~~under professional care. Our goal is to contribute a reproducible framework that can inform future multi-individual studies and enhance rehabilitation practices across institutions.

Materials and Methods

Ethical statement

This study was conducted in accordance with the Ethical Guidelines for the Conduct of Research on Animals by Zoos and Aquariums issued by the World Association of Zoos and Aquariums (WAZA) and the Code of Ethics established by the Japanese Association of Zoos and Aquariums (JAZA). It also adhered to the Animal Care Guidelines of the Okinawa Churashima Foundation, which oversees the Okinawa Churaumi Aquarium. Additionally, the study was approved by the Animal Experiment Committee established by the Okinawa Churashima Foundation (Permission Number: AT22003). These guidelines and committee oversight emphasize the importance of a thorough ~~proper~~ understanding and careful management of animal welfare. The acts involved in this study did not negatively impact the welfare of the dolphins involved.

Formatted: Font: 12 pt

Formatted: Line spacing: Double

Formatted: Font: 12 pt

Formatted: Font: 12 pt

Formatted: Font: 12 pt

Subject Dolphin

The subject of this study was Sami, a captive-born adult female Indo-Pacific bottlenose dolphin (*Tursiops aduncus*) housed at the Okinawa Churaumi Aquarium. In February 2021, she underwent surgical amputation of the tail flukes due to trauma-induced necrosis. The rehabilitation process enabled a structured evaluation of prosthetic-assisted recovery in cetaceans with severe caudal injury, focusing on swimming function, social reintegration, and overall welfare.

Formatted: Font: 12 pt

In September 2020, the subject dolphin sustained a significant blunt trauma to her tail flukes after colliding with ~~the edge of~~ the pool wall. The resulting injury developed into a complex infection, leading to progressive necrosis of the affected tissue. On February 2021, post-injury, the tail flukes were surgically amputated, leaving only the central portion intact. The impact also caused a rupture of the ventral tendon on the right side of the caudal vertebrae, resulting in an asymmetrical deformation of the tail. Specifically, the distal portion of the vertebrae became twisted upward and to the right~~ward~~ (Fig~~.~~ 1).

Formatted: Font: 12 pt

Formatted: Font: 12 pt

Formatted: Font: 12 pt

Fig 1. Tail flukes amputation and prosthetic application in the Indo-Pacific bottlenose dolphin. (A) Lateral view of the subject dolphin following surgical removal

of the tail flukes due to trauma-induced necrosis. (B) Close-up of the residual tail stump showing asymmetrical deformation. (C) Custom-designed prosthetic tail flukes developed for attachment to the caudal peduncle. (D) The dolphin swimming with the prosthetic tail flukes attached, exhibiting vertical tail-beat motion.

Formatted: Font: 12 pt
Formatted: Line spacing: Double

Following the completion of the surgical amputation in June 2021, the subject dolphin initially attempted to swim using a lateral (side-to-side) tail motion. However, this failed to generate sufficient propulsion, and her voluntary swimming activity gradually declined. At the same time, her engagement in social interactions with other dolphins also decreased markedly. This state persisted for approximately one month. At the time of the incident, the subject dolphin measured 237 cm in length and weighed 143 kg.

Rehabilitation 1: Restoration of Swimming Ability Using a Prosthetic Tail flukes

In line with the case of Fuji, a Pacific bottlenose dolphin at our aquarium whose quality of life (QOL) was significantly improved through the use of prosthetic tail flukes [2], this study prioritized the restoration of swimming ability through the application of a prosthetic device. The subject dolphin had an asymmetrical distal end of the tail stump,

Formatted: Font: 12 pt
Formatted: Indent: First line: 0.5 ch, Line spacing: Double

requiring a secure attachment system capable of withstanding the forces generated during propulsion. A socket-type attachment was designed to encase the entire caudal peduncle, onto which rubber prosthetic tail flukes—shaped to resemble natural flukes—w~~as~~ere mounted (Fig~~.~~ 1, S1 Video).

Following the loss of her tail flukes, the subject dolphin developed a compensatory swimming pattern characterized by pronounced lateral body undulation (~~S1~~ S2 Video). While this strategy may have enabled limited locomotion, it imposed abnormal strain on muscle groups and tendons not typically engaged during normal cetacean swimming. To mitigate the risk of injury and to support functional recovery, rehabilitation efforts focused on reestablishing a species-typical stroke pattern involving vertical tail movements, which are essential for efficient propulsion and overall biomechanical balance.

To address this, a rehabilitation program was implemented between July 2021 and May 2022 to increase vertical tail flexibility and restore an appropriate stroke axis. During these sessions, the pool was drained and ~~her~~the dolphin's body was gently restrained. A single trainer manually guided the distal tail region through a full vertical range of motion—up and down—until natural resistance was encountered. Each session lasted approximately 20 minutes and was conducted ~~1~~ one to ~~5~~ five times per week, for a total

Formatted: Font: 12 pt

Formatted: Font: 12 pt

Formatted: Font: 12 pt

Formatted: Font: 12 pt

Formatted: Font: 12 pt

Formatted: Font: 12 pt

Formatted: Font: 12 pt

of 30 sessions (~~S2~~ S3 Video). No aversive or avoidance behaviors were observed during this phase.

Following this stretching rehabilitation, habituation training to the prosthetic device was carried out between May and December 2022. The prosthetic tail was attached, and the subject dolphin was allowed to swim freely in her exhibit pool for 5 to 15 minutes per session. This training was conducted 1 to 5 times per week for a total of 43 sessions. The goal of this phase was to promote physical adaptation and establish a positive behavioral association with the prosthesis.

Evaluation of Swimming Efficiency Using Prosthetic Tail flukes

To evaluate the effect of a prosthetic tail on swimming performance, we compared three conditions: (1) a dolphin without tail flukes (the subject dolphin), (2) the same dolphin with prosthetic tail flukes, and (3) a healthy individual of the same species. In all trials, the dolphins were encouraged to swim at maximum effort along the perimeter of ~~a~~ the pool. Swimming efficiency was assessed using data recorded by animal-borne data loggers.

The loggers used were OR1400-3MPD3GT ~~models~~ units manufactured by Little

Formatted: Font: 12 pt

Formatted: Line spacing: Double

Formatted: Font: 12 pt

Formatted: Indent: First line: 0.5 ch, Line spacing: Double

Formatted: Font: 12 pt

Formatted: Font: 12 pt

Formatted: Font: 12 pt

Leonardo Corporation. Two loggers were attached to each dolphin using suction cups—one placed just below the dorsal fin to record swim speed, and the other near the tail region to measure stroke frequency based on the periodicity of acceleration along the z-axis.

Data loggers recorded swim speed, stroke frequency, and tri-axial acceleration (x, y, and z axes). In the present analysis, the periodicity of acceleration along the longitudinal (z) axis was used to calculate stroke frequency, while swim speed was obtained from the synchronized data.

For the subject dolphin, nine trials were conducted without the prosthetic tail flukes and eight trials with it. In addition, nine trials were conducted in total ~~for~~ with two healthy Indo-Pacific bottlenose dolphins. For each trial, we extracted one complete tail stroke corresponding to the moment of maximum swim speed for analysis.

Swimming efficiency was quantified using the ~~Relative~~ relative ~~Propulsion~~ propulsion ~~Distance~~ distance per ~~Stroke~~ stroke (RPDS), a dimensionless index defined as:

~~RPDS = Swim Speed (m/s) / Body Length (m) / Stroke Frequency (stroke/s)~~

$$\mathrm{RPDS} = \frac{\mathrm{Swim\ \ speed\ \ (m\ \ s^{-1})}}{\mathrm{Body\ \ length\ \ (m)} \times \mathrm{Stroke\ \ frequency\ \ (storke\ \ s^{-1})}}$$

This index represents the relative distance traveled per stroke, normalized by body

Formatted: Font: (Default) Times New Roman, 12 pt

Formatted: Font: (Default) Times New Roman, 12 pt

Formatted: Font: 12 pt

Formatted: Font: 12 pt

Formatted: Font: 12 pt

Formatted: Font: 12 pt

Formatted: Font: 12 pt

Formatted: Font: 12 pt

Formatted: Font: Times New Roman, Not Italic

Formatted: Font: 12 pt

Formatted: Indent: First line: 0 ch, Line spacing: Double

Formatted: Indent: First line: 0.5 ch, Line spacing: Double

length and stroke frequency. Bainbridge [9] demonstrated a strong relationship between swim speed, body length, and tail beat frequency, supporting the validity of these parameters. The RPDS was therefore considered a particularly suitable indicator for evaluating and comparing the swimming efficiency of a dolphin without tail flukes, one equipped with prosthetic tail flukes, and healthy conspecifics.

However, the evaluation of swimming efficiency using the prosthetic tail was discontinued in January 2023 to prevent the progression of a localized infection observed at the distal end of the tail stump. This decision was made as a precautionary medical measure, and the prosthetic tail has not been used since, ~~then~~ to allow for proper treatment and healing.

Statistical comparisons of locomotor parameters among the rehabilitation phases were conducted using one-way analysis of variance (ANOVA) followed by Tukey's honest significant difference (HSD) post hoc test. All analyses were performed in Python (version 3.10) using standard scientific libraries.

Rehabilitation 2: Restoration of Social Behavior

Social relationships are known to play a critical role in the welfare of Indo-Pacific bottlenose dolphins [5,6]. Following the surgical removal of the tail flukes, the subject

Formatted: Font: 12 pt

Formatted: Line spacing: Double

Formatted: Font: 12 pt

Formatted: Font: 12 pt

Formatted: Font: 12 pt

Formatted: Indent: First line: 0.5 ch, Line spacing: Double

Formatted: Font: 12 pt

dolphin was housed alone throughout Rehabilitation Phase 1, which focused on restoring swimming ability. During this period, a short-term cohabitation trial was conducted with a familiar conspecific that had previously lived with the subject dolphin. The aim of this trial was to promote the reacquisition of natural social behaviors. However, the attempt was discontinued due to the emergence of aggressive behaviors, such as chasing and physical contact directed toward the subject dolphin, as she was unable to synchronize her movements with the healthy individual.

As Rehabilitation Phase 1 progressed, the subject dolphin began to exhibit~~ing~~ consistent, voluntary swimming characterized by stable vertical tail movements, indicating a recovery of motivation. Based on this improvement, a second short-term cohabitation trial was implemented. This time, no aggressive or avoidance behaviors (e.g., chasing or withdrawal) were observed. In light of this outcome, a gradual reintroduction to social interactions was initiated as Rehabilitation Phase 2, with the goal of restoring social engagement.

Rehabilitation Phase 2 was conducted over a period of 53 days between September and October 2024. The subject dolphin was housed in a large outdoor pool (maximum diameter: 25 m; depth: 6 m; water volume: 1,750 m^3) together with a ~~varied~~ diverse group of conspecifics and related species, including *Tursiops adu*ncus, *Tursiops truncat*us,

Formatted: Font: 12 pt

Formatted: Font: 12 pt

Formatted: Font: 12 pt

Pseudorca crassidens, and *Steno bredanensis*—all female individuals with whom she had previously established affiliative relationships. To promote the restoration of sociality through natural interactions, no special treatment was given to the subject dolphin during the cohabitation period, and human intervention in inter-animal dynamics was deliberately avoided. The number of cohabiting individuals (excluding the subject dolphin) fluctuated throughout the rehabilitation period due to management requirements: four animals were present for 26 days, five for 22 days, three for 3 days, and six for 2 days. Qualitative behavioral observations were conducted five times daily, ~~in principle~~generally aligned with the five scheduled feeding sessions. These observations focused on spontaneous interactions, behavioral changes, and social dynamics among dolphins and served as a basis for evaluating the progression of her social reintegration in a naturalistic setting.

To quantitatively assess behavioral changes before and after Rehabilitation Phase 2, focal observations were conducted on the subject dolphin over two distinct periods: four days between November 2021 and January 2022 (pre-rehabilitation), and three days between October and November 2024 (post-rehabilitation). During the pre-rehabilitation period, the subject dolphin was housed with a female rough-toothed dolphin (*Steno bredanensis*) and bottlenose dolphins (*Tursiops truncatus*), while in the post-rehabilitation period, she

Formatted: Font: 12 pt

Formatted: Line spacing: Double

was cohabiting with female bottlenose dolphins and false killer whales (*Pseudorca crassidens*). All cohabiting animals had previously exhibited affiliative relationships with the subject dolphin prior to her injury. In both periods, cohabitation took place in an outdoor tank measuring 14 meters in length, 3 meters in depth, and with a water volume of 400 m³.

The focal animal's behavioral state was recorded every 5 minutes over a 1-hour period using scan sampling. Observations were consistently conducted from 12:30 to 13:30, a non-feeding period, to avoid the influence of feeding motivation. Because the behavioral state was recorded objectively at fixed 5-minute intervals using scan sampling, observer bias and recording errors were minimized. All sessions were conducted while the dolphin was not wearing ~~a~~ prosthetic tail flukes.

To evaluate the dolphin's psychological and social state, six behavioral categories were used, based on the definitions provided by Miller et al.[6]: resting, affiliative behavior, aggressive behavior, repetitive behavior, high-energy behavior, and others (e.g., routine swimming, object play, exploratory behavior). As a behavioral control, the same behavioral observations were also conducted on a healthy adult female Indo-Pacific bottlenose dolphin over seven days between October 2024 and January 2025. This individual ~~has~~ had been under human care at the Okinawa Churaumi Aquarium since

Formatted: Font: 12 pt

Formatted: Font: (Default) Times New Roman, 12 pt

Formatted: Font: 12 pt

Formatted: Font: 12 pt

Formatted: Font: 12 pt

1975 and ~~is~~ was the biological mother of the subject dolphin. While her exact age is unknown, her long-term residency and environmental familiarity were considered appropriate for use as a behavioral reference in this study.

Formatted: Font: 12 pt

Result

Rehabilitation Phase 1

Rehabilitation Phase 1, which focused on expanding the vertical range of tail motion and correcting the stroke axis from horizontal to vertical, required approximately 11 months to complete. As a result of this intervention, the subject dolphin regained the ability to swim with a consistent vertical tail beat. Habituation to the prosthetic tail flukes also progressed smoothly. Within approximately 3 months from the start of this phase, the subject dolphin demonstrated stable and coordinated swimming while wearing the prosthetic (~~S3~~ S4 Video). Notably, even when not wearing the prosthetic tail flukes, the subject dolphin no longer exhibited the snake-like lateral body undulation that was previously observed immediately following tail loss. This behavioral change suggests that the vertical tail stroke pattern was retained even in the absence of the prosthetic device, indicating the effectiveness of the rehabilitation in promoting lasting motor adaptation.

Formatted: Font: 12 pt

Formatted: Font: 12 pt

Swimming Efficiency Using Prosthetic Tail flukes

Fig~~ure~~ 2 illustrates the results of high-speed swimming trials under three conditions: without tail flukes (No Fin), with prosthetic tail flukes, and in a healthy control individual. The maximum swimming speed was significantly lower in both the no fin and prosthetic conditions compared to the control group ($p < 0.001$), with no significant difference between the no fin and Prosthetic groups.

Fig 2. Boxplots of maximum swim speed (A), stroke frequency (B), and relative propulsive distance per stroke (RPDS) (C) measured by accelerometer under three conditions: No Fin, Prosthetic Tail, and Control (a healthy individual with intact tail flukes). Asterisks indicate statistically significant differences between groups based on Tukey's HSD test ($p < 0.05$). Boxes represent the interquartile range (IQR), horizontal lines inside boxes indicate medians, and whiskers extend to 1.5×IQR. Outliers are plotted as individual points.

Stroke frequency, measured as the number of tail beats per second, did not differ significantly between the no fin and control groups ($p = 0.53$), but was significantly reduced in the prosthetic condition compared to both no fin and control ($p < 0.001$).

Formatted: Font: 12 pt

Formatted: Font: Bold

Formatted: Font: Bold

Formatted: Font: 12 pt

Formatted: Line spacing: Double

Formatted: Indent: First line: 0.5 ch, Line spacing: Double

Relative propulsion distance per stroke (RPDS), which quantifies body-length-normalized distance traveled per tail stroke, differed significantly among all three groups ($p < 0.001$). The no fin condition showed the lowest RPDS values, while the prosthetic condition was significantly higher and closer to Control levels. In some trials, RPDS values in the prosthetic condition were comparable to or even exceeded those observed in healthy individual.~~s~~

These results indicate that while the prosthetic tail did not contribute substantially to increasing swim speed—likely due to increased drag and reduced flexibility—it partially restored propulsion efficiency lost due to tail loss. Notably, even though stroke frequency was reduced in the prosthetic condition, the distance traveled per stroke was improved, supporting the effectiveness of the prosthetic tail as a functional assistive device for locomotion.

Since January 2023, the use of the prosthetic tail has been discontinued ~~based on~~for medical ~~considerations~~reasons. Nevertheless, until June 2025, when the individual died due to unrelated chronic conditions, primarily pulmonary in nature., the subject dolphin consistently maintained the vertical tail-beat swimming pattern acquired through rehabilitation, suggesting that the motor adaptation had been retained in the long-term.

Formatted: Font: 12 pt

Formatted: Font: 12 pt

Formatted: Font: 12 pt

Formatted: Font: 12 pt

Formatted: Font: 12 pt

Formatted: Line spacing: Double

Rehabilitation Phase 2

During Rehabilitation Phase 2, which aimed to restore social behaviors through active cohabitation, affiliative interactions began to emerge shortly after the initiation of the program. Behaviors such as synchronous swimming and close-contact positioning—completely absent prior to rehabilitation—were consistently observed. In addition, instances of drafting behavior—defined as one individual swimming in the hydrodynamic wake of another—were documented during cohabitation (S45 Video). As this behavior entails coordinated movement and sustained proximity, it is considered a form of affiliative interaction indicative of social cohesion. Quantitative behavioral analysis (Fig. 3) revealed that prior to rehabilitation, the subject dolphin spent most of the observation time resting motionlessly at the surface, with no affiliative or social interactions recorded. In contrast, near the end of Rehabilitation Phase 2, the proportion of resting behavior had markedly decreased, while the "Others" category nearly doubled. Notably, affiliative behaviors accounted for approximately 17% of total observed behaviors. Within the "Others" category, the majority consisted of solitary swimming, with a smaller portion comprising exploratory and solitary play behaviors.

Fig 3 Behavioral composition of a rehabilitated Indo-Pacific bottlenose dolphin

Formatted: Font: 12 pt

Formatted: Font: 12 pt

Formatted: Font: 12 pt

Formatted: Font: Bold

Formatted: Indent: First line: 0 ch

before and after rehabilitation, compared with a healthy control. Pie charts represent the proportion of six behavioral categories—resting, affiliative, high-energy, and others—observed during focal sampling sessions. “Others” includes routine swimming, solitary play, and exploratory behaviors.

Compared to a healthy adult *Tursiops aduncus* observed under similar conditions, the subject dolphin demonstrated more than ~~twice~~ double the frequency of affiliative behavior. No aggressive or repetitive behaviors were recorded throughout the observation period, suggesting that the subject dolphin had re-established social engagement in a stable and positive manner.

Remarkably, these affiliative behaviors—including synchronous swimming and close-contact positioning—were observed even during the final stage of her life, while she was affected by a pulmonary condition, continuing up to the day before her death.

Discussion

Ethical Considerations in Rehabilitation

Ethical considerations were integral to decision-making throughout the rehabilitation

Formatted: Font: 12 pt

Formatted: Indent: First line: 1 ch, Line spacing: Double

Formatted: Indent: First line: 0.5 ch, Line spacing: Double

Formatted: Font: 12 pt

Formatted: Font: 12 pt

Formatted: Line spacing: Double

Formatted: Line spacing: Double

Formatted: Font: (Default) Times New Roman, 16 pt, Bold

Formatted: Line spacing: Double

Formatted: Indent: First line: 0.5 ch, Line spacing: Double

process. The duration of prosthesis use was determined through continuous medical assessment and welfare monitoring, and the device was ultimately discontinued in accordance with the precautionary principle to prevent further infection at the stump site. Similarly, the timing of social reintroduction was guided by the dolphin’s behavioral stability, health status, and stress indicators to ensure optimal welfare conditions. These decisions were made collaboratively by veterinarians and animal keepers, reflecting a welfare-centered and precautionary approach to rehabilitation.

Evaluation of Physical Recovery and Social Behavior

The rehabilitation program implemented for the subject dolphin resulted in significant improvements in both her physical performance and social engagement. In terms of swimming ability, the structured training allowed her to recover a vertical tail-beat locomotion, which had been lost following the injury. Although swimming speed in both the No Fin and Prosthetic conditions remained significantly lower than in healthy dolphins (Fig. 2), the use of the prosthetic tail partially restored propulsion efficiency, as evidenced by a significantly higher RPDS compared to the No Fin condition. These findings indicate that while the prosthetic tail did not substantially improve speed, likely due to increased drag and reduced flexibility, it served as a functional assistive device that

Formatted: Line spacing: Double

Formatted: Font: 12 pt

Formatted: Indent: First line: 0.5 ch, Line spacing: Double

Formatted: Font: 12 pt

improved swimming efficiency per stroke. Notably, the subject dolphin continued to exhibit a stable vertical tail-beat pattern even after the prosthesis had been discontinued due to medical considerations, suggesting lasting motor adaptation.

In the domain of social behavior, progress was even more striking. At the end of Rehabilitation Phase 2, affiliative behaviors—such as synchronous swimming and close-contact positioning—accounted for approximately 17% of total observed behavior (Fig. 3). Prior to rehabilitation, such interactions were entirely absent, and the subject dolphin spent nearly all her time resting motionlessly at the surface. In contrast, post-rehabilitation observations showed a substantial reduction in resting and an increase in active behaviors, including solitary swimming, exploratory movement, and solitary play. Within the "Others" category, the majority consisted of solitary swimming, with a smaller portion comprising exploratory and solitary play behaviors.

Moreover, her social activity time fell within the range observed in wild dolphins, which spend approximately 4–31% of their time engaged in social behaviors [6]. Compared ~~to~~ with a healthy adult *Tursiops aduncus* observed under similar conditions, the subject dolphin demonstrated more than ~~twice~~ double the frequency of affiliative behavior. Her successful reintegration into the social group underscores the rehabilitation program's effectiveness in addressing both physical and social challenges, and it contributed

Formatted: Font: 12 pt

Formatted: Font: 12 pt

Formatted: Font: 12 pt

Formatted: Font: 12 pt

significantly to her overall recovery and quality of life (QOL). Notably, both the vertically undulating swimming pattern and affiliative behaviors were retained until the final stage of her life, even after the progression of a fatal pulmonary condition. The long-term retention of vertical locomotion and affiliative behaviors indicates the potential for sustained adaptation following targeted rehabilitation interventions.

Psychological Changes and Resilience

Beyond these physical and behavioral outcomes, the rehabilitation process appeared to foster notable psychological changes. The repeated success in adapting to the prosthetic tail flukes, followed by the maintenance of learned motor patterns even after its discontinuation, may be indicative of enhanced self-efficacy—the belief in one's ability to perform essential tasks [10]. This concept, originally developed in human psychology, has gained increasing relevance in the context of animal rehabilitation, particularly for cognitively advanced species such as dolphins [11].

Another critical factor in her recovery was the successful generalization of learned behaviors and the restoration of a species-typical movement pattern. Skills acquired during prosthetic-assisted swimming—such as vertical tail-beat motion and coordinated body control—were effectively transferred to situations ~~in which~~where the prosthesis was

Formatted: Line spacing: Double

Formatted: Font: 12 pt

Formatted: Indent: First line: 0.5 ch, Line spacing: Double

Formatted: Font: 12 pt

Formatted: Font: 12 pt

Formatted: Font: 12 pt

Formatted: Font: 12 pt

no longer used. This continuity suggests the development of robust motor learning and supports Shepard's theory of generalization [12], whereby behaviors learned in one condition can be applied to analogous situations through shared psychological processes. Importantly, the regained vertical tail movement may have played a key role not only in sustaining physical mobility but also in facilitating social reintegration. It is plausible that movement patterns consistent with species-typical norms contribute to social recognition and acceptance within animal groups. In some mammalian species, disruptions to such behavioral cues have been linked to social rejection or exclusion. For instance, chimpanzees that exhibit aberrant or socially inappropriate behavior are known to be actively shunned by group members [13], and African elephants that experience~~d~~ early-life social disruption have shown non-normative behavior and difficulties in reintegrating into social groups due to deficits in learned social and motor patterns [14]. Furthermore, abnormal locomotor patterns—especially those that deviate from species-typical mechanics—may trigger avoidance or aggression from peers [15] By regaining vertical tail-beat locomotion, the subject dolphin may have reduced the perceptual incongruence that could otherwise have led to negative social responses, ultimately promoting social acceptance and the emergence of affiliative behaviors during cohabitation.

Formatted: Font: 12 pt

Formatted: Font: 12 pt

Formatted: Font: 12 pt

Formatted: Font: 12 pt

Formatted: Font: 12 pt

The Role of Positive Reinforcement

Throughout both rehabilitation phases, positive reinforcement played a central role. Reward-based training not only facilitated prosthetic adaptation but also reinforced a positive association with swimming and physical engagement. The continuation of voluntary swimming and exploration after the discontinuation of the prosthesis further suggests that intrinsic motivation ~~was~~ had been established. Parallels can be found in human and non-human primate rehabilitation, where structured positive reinforcement has been shown to enhance motivation, reduce stress, and improve adaptability [16,17]. These findings underscore the cross-species relevance of reinforcement-based methodologies in promoting behavioral resilience and psychological well-being.

Implications for Animal Welfare and Rehabilitation

These findings emphasize the importance of a holistic approach to rehabilitation that addresses both physical and psychological domains. Reintegration into a social group was a critical component of recovery, as social affiliation and culturally transmitted behaviors are vital to the well-being of cetaceans [18]. In highly social species like the Indo-Pacific bottlenose dolphin, providing opportunities for meaningful social engagement is essential for long-term welfare. The observed resilience and adaptability further highlight the role

Formatted: Line spacing: Double

Formatted: Font: 12 pt

Formatted: Indent: First line: 0.5 ch, Line spacing: Double

Formatted: Font: 12 pt

Formatted: Font: 12 pt

Formatted: Line spacing: Double

Formatted: Font: 12 pt

Formatted: Font: 12 pt

Formatted: Font: (Default) Times New Roman, 12 pt

Formatted: Font: 12 pt

of cognitive processing in animal welfare, as discussed by Duncan ~~&~~ and Petherick [15]. Structured rehabilitation programs that incorporate opportunities for learning, problem-solving, and autonomous interaction can enhance not only physical recovery but also psychological health and behavioral flexibility.

Although prosthetic applications in marine mammals other than dolphins have not been reported, similar attempts have been made in sea turtles that have lost their flippers. These studies, however, have largely focused on the engineering and hydrodynamic aspects of prosthetic design rather than on behavioral or welfare outcome [19,20]~~{Citation}~~. In contrast, the present study demonstrates how prosthetic technology can be integrated into a welfare-centered rehabilitation program that simultaneously addresses locomotor function, social behavior, and psychological recovery.

~~Finally, t~~The observed outcomes underscore the value of structured, welfare-centered interventions in promoting recovery among cetaceans with severe injuries. The use of multidimensional metrics—including activity patterns, social behavior, and adaptive functioning—may support more standardized and species-appropriate assessments of rehabilitation success. Such frameworks could improve not only the design of future interventions but also transparency and accountability in animal welfare practices [21].

Although the subject dolphin showed sustained behavioral stability and social

Formatted: Font: 12 pt

Formatted: Font: 12 pt

Formatted: Font: (Default) Times New Roman, 12 pt

Formatted: Font: (Default) Times New Roman, 12 pt

Formatted: Indent: First line: 0.5 ch, Line spacing: Double

Formatted: Font: (Default) Times New Roman, 12 pt

Formatted: Font: (Default) Times New Roman, 12 pt

Formatted: Font: (Default) Times New Roman, 12 pt

Formatted: Font: (Default) Times New Roman, 12 pt

Formatted: Font: 12 pt

Formatted: Font: 12 pt

Formatted: Font: 12 pt

Formatted: Font: 12 pt

Formatted: Font: (Default) Times New Roman, 12 pt

Formatted: Indent: First line: 0.5 ch, Line spacing: Double

engagement following rehabilitation, ensuring long-term welfare remains crucial when individuals have undergone severe physical trauma. Continuous assessment of physical condition, social relationships, and behavioral diversity can help detect subtle signs of discomfort, social isolation, or chronic stress. Welfare frameworks such as the Five Domains model [4] highlight the need for multidimensional evaluation and management responsive to each animal's mental state. Moreover, recent reviews of marine mammal rehabilitation emphasize the paucity of robust long-term post-release and welfare data [8], reinforcing the imperative for ongoing monitoring. Adaptive management—guided by systematic welfare data—allows caretakers to modify group composition, enrichment programs, or medical interventions in response to changing welfare needs. Establishing longitudinal welfare datasets across facilities would further enhance our understanding of recovery trajectories and support evidence-based refinement of rehabilitation practices for marine mammals.

Practical Guidelines for Dolphin Rehabilitation

Recent global reviews have highlighted the lack of standardized clinical frameworks and quantitative welfare assessments in marine mammal rehabilitation programs [8]. This study presents a structured, welfare-centered protocol that directly addresses this

Formatted: Font: (Default) Times New Roman, 12 pt

Formatted: Font: (Default) Times New Roman, 12 pt

Formatted: Font: (Default) Times New Roman, 12 pt

Formatted: Font: (Default) Times New Roman, 12 pt

Formatted: Font: 12 pt

Formatted: Font: (Default) Times New Roman, 16 pt, Bold

Formatted: Line spacing: Double

Formatted: Font: (Default) Times New Roman

Formatted: Font: (Default) Times New Roman

gap, providing a practical example of how standardization can be implemented in real-world settings. The experience from this case offers practical insights for facilities managing dolphins with severe caudal injuries.

A two-phase rehabilitation framework—comprising physical and social recovery—proved effective in guiding clinical decisions and monitoring progress.[7]

During Phase 1 (physical rehabilitation), wound healing and muscle recovery should be prioritized, and the timing of prosthesis introduction should be determined cautiously, considering infection risk and skin tolerance.

During Phase 2 (social rehabilitation), gradual reintroduction to conspecifics and careful behavioral monitoring are essential to ensure social stability and psychological well-being.

Establishing objective behavioral and locomotor indicators to assess progress at each phase would further strengthen the reproducibility of such approaches. These recommendations could serve as a practical foundation for developing future standardized clinical protocols in marine mammal rehabilitation.

Implementation of such rehabilitation programs requires coordinated collaboration among veterinarians, animal trainers, and engineers to ensure safety and welfare at each stage. Staff should receive specialized training in prosthetic handling, behavioral

Formatted: Font: (Default) Times New Roman

Formatted: Font: (Default) Times New Roman

Formatted: Font: Times New Roman, 12 pt

Formatted: Font: (Default) +Body (Yu Mincho)

Formatted: Font: Not Bold

Formatted: Font: (Default) +Body (Yu Mincho)

Formatted: Font: Not Bold

Formatted: Font: (Default) +Body (Yu Mincho)

Formatted: Font: (Default) +Body (Yu Mincho)

Formatted: Font: (Default) +Body (Yu Mincho)

Formatted: Font: (Default) +Body (Yu Mincho)

Formatted: Font: (Default) +Body (Yu Mincho)

Formatted: Font: (Default) Times New Roman, 12 pt

Formatted: Normal, Indent: First line: 0.5 ch, Space Before: Auto, After: Auto, Line spacing: Double

Formatted: Font: (Default) Times New Roman, 12 pt

monitoring, and welfare assessment to minimize stress during interventions. Facilities must be equipped with adaptable pools that allow controlled water depth for physical therapy, as well as access to materials and fabrication expertise for custom prosthetic design. Although initial development costs—such as prosthetic fabrication and data-logging equipment—can be considerable, these are often offset by the long-term welfare benefits and improved management efficiency achieved through structured rehabilitation. Sharing technical resources and training programs among institutions would help reduce costs and promote wider adoption of welfare-centered rehabilitation frameworks.

Conclusions

This study evaluated the outcomes of a structured rehabilitation program for a tail-fluke-amputated Indo-Pacific bottlenose dolphin through a structured, multi-phase program aimed at restoring swimming ability, social integration, and psychological well-being. We hypothesized that a welfare-centered intervention combining prosthetic-assisted locomotor training and socially enriching environments could lead to long-term functional and behavioral recovery. Our findings support this hypothesis.

The use of custom-made prosthetic tail flukes significantly improved propulsion per

Formatted: Font: (Default) Times New Roman, 12 pt

Formatted: Font: (Default) Times New Roman, 12 pt

Formatted: Font: (Default) Times New Roman, 12 pt

Formatted: Font: (Default) Times New Roman, 12 pt

Formatted: Font:

Formatted: Font: 12 pt

Formatted: Line spacing: Double, Tab stops: 1.05", Left

Formatted: Line spacing: Double

Formatted: Font: 12 pt

Formatted: Indent: First line: 0.5 ch, Line spacing: Double

stroke (RPDS), even though absolute swim speed remained suboptimal due to mechanical constraints. Notably, vertical tail-beat locomotion was retained even after prosthesis discontinuation, indicating durable motor adaptation. Socially, affiliative behavior increased to levels surpassing those observed in healthy conspecifics, with no aggression or abnormal behaviors recorded, suggesting a complete reintegration into the social group. These improvements were sustained until the final stage of life, highlighting the lasting impact of rehabilitation.

This study contributes new insights to the field of marine mammal welfare by (1) providing quantitative evidence of long-term biomechanical and social adaptation following tail loss, (2) demonstrating the value of prosthetic training beyond physical function, including psychological resilience and enhanced self-efficacy, and (3) proposing a framework that integrates physical, social, and psychological metrics for evaluating rehabilitation success.

Limitations of this study include its single-subject design and the inability to assess long-term prosthetic use due to medical decisions aimed at preventing the progression of infection. While these constraints are inherent to case-based studies, they also highlight the need for broader, collaborative efforts to validate and extend the present findings. To overcome the limitations of single-case studies, future research should involve multi-

Formatted: Font: 12 pt

Formatted: Font: (Default) Times New Roman, 12 pt, Not Bold

Formatted: Font: (Default) Times New Roman, 12 pt

institutional collaborations that allow comparative and longitudinal assessments of prosthetic adaptation and social reintegration. Establishing shared behavioral metrics and rehabilitation records across facilities would enable the identification of individual variability and key factors for success, contributing to a more generalizable welfare assessment framework. Such collaborative efforts will strengthen the scientific basis of marine mammal rehabilitation and promote higher international standards of welfare practice.~~Future research should explore the scalability of such interventions to other cases and species, refine prosthetic materials to enhance tolerance, and investigate cognitive and emotional outcomes in rehabilitated animals.~~ It is also worth speculating that improved motor patterns may have facilitated social acceptance by reducing visual incongruence—a hypothesis that warrants further ethological investigation.

Taken together, this study underscores the feasibility and necessity of holistic rehabilitation approaches for cetaceans with severe physical trauma. By addressing mobility, sociality, and emotional health in tandem, such programs can meaningfully improve quality of life and serve as a model for welfare-driven innovation in marine animal care.

Acknowledgement

Formatted: Font: (Default) Times New Roman, 12 pt

Formatted: Font: 12 pt

Formatted: Line spacing: Double

We thank the staff at the Okinawa Churaumi Aquarium for animal training, handling, and medical treatment. Koji Tokutake and Dr. Isao Kawazu, former managers of the marine mammal department, provided encouragement and support throughout this project. We also thank Bridgestone Corporation for their generous financial and resource support in the development of the prosthetic tail. We are also grateful to the staff of the Wildlife Research Center of Kyoto University for kindly providing the data loggers used to measure swimming performance. We also express our sincere respect to Sami, the Indo-Pacific Bottlenose Dolphin who participated in this study. Although she passed away due to a lung disease during the manuscript preparation, her contributions to our understanding of physical and social recovery in marine mammals remain invaluable. Artificial intelligence assistance (ChatGPT, OpenAI) was used solely for language polishing and improving the clarity of the English text. All scientific content, data analysis, and conclusions were developed entirely by the authors.

Formatted: Font: 12 pt

Formatted: Font: Not Bold

Formatted: Font: 12 pt

References

1. Miller L, Mellen J, Greer T, Ii SK. The effects of education programmes on Atlantic bottlenose dolphin *(Tursiops truncatus)* behaviour. Anim welf. 2011;20: 159–172. doi:10.1017/S0962728600002645

2. Ueda K, Murakami M, Kato J, Miyahara H, Izumisawa Y. Intervention to improve the quality of life of a Bottlenose dolphin that developed necrosis on the tail flukes. J Phys Ther Sci. 2013;25: 1201–1207. doi:10.1589/jpts.25.1201

3. Kagan R, Carter S, Allard S. A. Universal animal welfare framework for zoos. Journal of Applied Animal Welfare Science. 2015;18: S1–S10. doi:10.1080/10888705.2015.1075830

4. Mellor DJ, Beausoleil NJ, Littlewood KE, McLean AN, McGreevy PD, Jones B, et al. The 2020 Five domains model: Including human–animal interactions in assessments of animal welfare. Animals. 2020;10: 1870. doi:10.3390/ani10101870

5. Lauderdale LK, Mellen JD, Walsh MT, Granger DA, Miller LJ. Towards understanding the welfare of cetaceans in accredited zoos and aquariums. Zaccaroni A, editor. PLoS ONE. 2021;16: e0255506. doi:10.1371/journal.pone.0255506

6. Miller LJ, Lauderdale LK, Mellen JD, Walsh MT, Granger DA. Relationships between animal management and habitat characteristics with two potential indicators of welfare for bottlenose dolphins under professional care. Li S, editor. PLoS ONE. 2021;16: e0252861. doi:10.1371/journal.pone.0252861

7. Clearwater Marine Aquarium. Winter the dolphin. n.d. Available: https://www.cmaquarium.org/animals/dolphins/winter/

8. Simeone CA, Rousselet E, Atkin C, De Trez M, Delemotte M, Johnson SP. A systematic review of global marine mammal rehabilitation and refloating, 2000–2023. Sustainability. 2024;16: 4589. doi:10.3390/su16114589

9. Bainbridge R. The speed of swimming of fish as related to size and to the frequency and amplitude of the tail beat. Journal of Experimental Biology. 1958;35: 109–133. doi:10.1242/jeb.35.1.109

10. Bandura A. Self-efficacy: Toward a unifying theory of behavioral change. Psychological Review. 1977;84: 191–215. doi:10.1037/0033-295X.84.2.191

11. Marino L. Convergence of complex cognitive abilities in cetaceans and primates. Brain Behav Evol. 2002;59: 21–32. doi:10.1159/000063731

12. Shepard RN. Toward a universal law of generalization for psychological science. Science. 1987;237: 1317–1323. doi:10.1126/science.3629243

13. Goodall J. The chimpanzees of Gombe: patterns of behavior. Cambridge, Massachusetts London, England: The Belknap Press of Harvard University Press; 1986.

14. Shannon G, Slotow R, Durant SM, Sayialel KN, Poole J, Moss C, et al. Effects of social disruption in elephants persist decades after culling. Front Zool. 2013;10: 62. doi:10.1186/1742-9994-10-62

15. Duncan IJ, Petherick JC. The implications of cognitive processes for animal welfare. Journal of Animal Science. 1991;69: 5017–5022. doi:10.2527/1991.69125017x

16. Ryan RM, Deci EL. Self-determination theory and the facilitation of intrinsic motivation, social development, and well-being. American Psychologist. 2000;55: 68–78. doi:10.1037/0003-066X.55.1.68

17. Laule G, Whittaker M. Enhancing nonhuman primate care and welfare through the use of positive reinforcement training. Journal of Applied Animal Welfare Science. 2007;10: 31–38. doi:10.1080/10888700701277311

18. Rendell L, Whitehead H. Culture in whales and dolphins. Behav Brain Sci. 2001;24: 309–324. doi:10.1017/S0140525X0100396X

19. Van Der Geest N, Garcia L. Employing robotics for the biomechanical validation of a prosthetic flipper for sea turtles as a substitute for animal clinical trials. Biomechanics. 2023;3: 401–414. doi:10.3390/biomechanics3030033

20. Sun X, Kato N, Matsuda Y, Kanda K, Kosaka Y, Kamezaki N, et al. Three-dimensional hydrodynamic analysis of forelimb propulsion of sea turtle with prosthetic flippers. J Abmech. 2013;3: 36–44. doi:10.5226/jabmech.3.36

21. Scott EM, Nolan AM, Reid J, Wiseman-Orr ML. Can we really measure animal quality of life? Methodologies for measuring quality of life in people and other animals.

Animal Welfare. 2007;16: 17–24.

Supporting information

S1_Video. This supplementary video illustrates the structure and attachment procedure of the artificial tail fluke used during Phase 2 of rehabilitation. The prosthesis consisted of a rubber tail fluke connected to a carbon fiber socket via a metal plate. The socket was divided into upper and lower sections, which were joined and secured with bolts. A silicone lining was inserted inside the socket to prevent abrasion of the caudal peduncle and to improve comfort and safety during attachment.

~~S1~~**S2_Video.** Video recording of the subject dolphin exhibiting pronounced lateral body undulation following tail fluke loss. Although a prosthetic tail is attached, the vertical tail-beat motion had not yet been adequately rehabilitated. As a result, the observed swimming behavior deviates from the typical pattern and imposes increased biomechanical strain on the axial body musculature.

~~S2~~**S3_Video.** Video footage depicting the rehabilitation procedure aimed at restoring vertical tail-beat motion. As described in the main text, the dolphin's body is gently

Formatted: Font: 12 pt
Formatted: Line spacing: Double
Formatted: Line spacing: Double
Formatted: Font: (Default) Times New Roman, 12 pt
Formatted: Font: (Default) Times New Roman, 12 pt
Formatted: Font: (Default) Times New Roman, 12 pt, Not Bold
Formatted: Font: (Default) Times New Roman, 12 pt
Formatted: Font: (Default) Times New Roman, 12 pt, Not Bold
Formatted: Font: (Default) Times New Roman, 12 pt
Formatted: Font: (Default) Times New Roman, 12 pt, Not Bold
Formatted: Font: (Default) Times New Roman, 12 pt
Formatted: Font: (Default) Times New Roman, 12 pt
Formatted: Font: (Default) Times New Roman, 12 pt, Not Bold
Formatted: Font: (Default) Times New Roman, 12 pt
Formatted: Font: (Default) Times New Roman, 12 pt, Not Bold
Formatted: Font: (Default) Times New Roman, 12 pt
Formatted: Font: (Default) Times New Roman, 12 pt
Formatted: Font: (Default) Times New Roman, 12 pt, Not Bold
Formatted: Font: (Default) Times New Roman, 12 pt
Formatted: Font: (Default) Times New Roman, 12 pt, Not Bold
Formatted: Font: (Default) Times New Roman, 12 pt
Formatted: Font: (Default) Times New Roman, 12 pt, Not Bold
Formatted: Font: (Default) Times New Roman, 12 pt
Formatted: Font: 12 pt
Formatted: Line spacing: Double
Formatted: Font: 12 pt
Formatted: Font: 12 pt
Formatted: Font: 12 pt

restrained while a trainer manually guides the distal tail region through its full vertical range of motion to promote flexibility and reestablish proper stroke axis.

S3S4 **Video.** Video recording of the subject dolphin exhibiting stable and coordinated swimming while wearing the prosthetic tail flukes. The footage illustrates successful habituation to the device, achieved during Rehabilitation Phase 2.

Formatted: Font: 12 pt

S45 **Video.** Video footage of the subject dolphin engaged in affiliative social behaviors after Rehabilitation Phase 2. The clip shows consistent synchronous swimming and close-contact positioning with conspecifics, as well as an instance of drafting behavior—defined as swimming in the hydrodynamic wake of another dolphin. These behaviors reflect the reestablishment of social cohesion and affiliative engagement following rehabilitation.

Formatted: Font: 12 pt

S56 **Table.** The raw data used to generate the figures and statistical results in the manuscript are provided as a supplementary file. This includes behavioral observations and swimming performance measurements necessary to replicate the analyses.

Formatted: Font: 12 pt

Response to the reviewer's comments

We would like to resubmit our manuscript entitled "Rehabilitation outcomes following tail-fluke amputation in an Indo-Pacific bottlenose dolphin: a welfare-centered approach," which has been revised in accordance with the valuable comments provided by the reviewers and the editor. In addition to addressing each point in detail, as outlined in the response table below, we have also implemented several general revisions to enhance the overall quality of the manuscript and to ensure full compliance with the PLOS ONE submission requirements. Specifically, the entire manuscript has been reformatted to conform to the journal's guidelines, including adjustments to the title page structure, author affiliations, heading levels, figure and table citations, file naming conventions, and reference style.

Reviewer comments	Answer comments
**Literature Review Currency:** The reference list requires substantial updating, with only 11% of references from the last five years. Incorporate recent publications (2020-2024) in marine mammal rehabilitation, prosthetics, and animal welfare science to better contextualize your work.	We have updated the reference list to include the most relevant and recent works in marine mammal welfare and rehabilitation. Consequently, the proportion of references published within the past five years has increased from about 11% to 24% (5 of 21 references). Because comprehensive rehabilitation studies in marine mammals are still at an early stage, peer-reviewed research directly addressing prosthetic-assisted rehabilitation remains scarce. This study therefore integrates the latest available evidence and provides pioneering insights that may guide future welfare-centered rehabilitation efforts.
**Comparative Analysis Enhancement:** While the current comparison is adequate, strengthen the discussion by comparing your findings with other marine mammal rehabilitation cases and recent advances in wildlife prosthetics.	We have expanded the *Discussion* to include reference to prosthetic interventions in other aquatic animals. Specifically, we mentioned studies on sea turtles that focused on the engineering and hydrodynamic design of prosthetic flippers. This addition clarifies the study's originality by contrasting previous technically oriented attempts with the welfare-

	centered rehabilitation framework applied to a dolphin.
**Methodological Detail:** Provide more comprehensive information about assessment protocols, including specific metrics used in biologging analysis, behavioral coding systems, and inter-observer reliability measures.	We have revised the *Materials and Methods* section to include additional methodological details. Specifically, we added information describing the parameters recorded by the biologging devices (swim speed, stroke frequency, and tri-axial acceleration) and clarified how these variables were analyzed. In addition, we included a statement that behavioral observations were conducted objectively at fixed 5-minute intervals using scan sampling, which minimized observer bias and recording errors. These additions enhance the transparency and reproducibility of the assessment protocols.
**Long-term Monitoring:** Expand the discussion of long-term welfare considerations, including recommendations for ongoing monitoring and potential adaptive management strategies.	We added a new paragraph in the *Discussion* to address long-term welfare considerations. The revised text emphasizes the importance of continuous monitoring, adaptive management, and data sharing for sustained welfare outcomes, citing the Five Domains framework and recent reviews of marine mammal rehabilitation.
**Resource Implications:** Provide more detailed discussion of the practical implementation requirements, including staff training, facility needs, and cost considerations for other institutions considering similar approaches.	We have added a new paragraph in the Practical Guidelines for Dolphin Rehabilitation section to address resource and implementation considerations. The new text discusses staff training requirements, facility specifications, and cost implications, and highlights how institutional collaboration can improve feasibility and reduce expenses.
**Ethical Considerations:** Enhance the discussion of ethical decision-making processes	We have added a new paragraph titled "Ethical Considerations in Rehabilitation" before the

throughout the rehabilitation, particularly regarding prosthesis use duration and social reintroduction timing.	Evaluation of Physical Recovery and Social Behavior section. The added text describes how the duration of prosthesis use and the timing of social reintroduction were determined through medical evaluation, welfare monitoring, and behavioral assessment, emphasizing a welfare-centered and precautionary decision-making process.
**Statistical Analysis:** Provide clearer explanation of any statistical methods used in analyzing behavioral and locomotor data, including effect size measures where appropriate.	We have clarified the statistical analysis in the *Evaluation of Swimming Efficiency Using Prosthetic Tail Flukes* section. Specifically, we added a description stating that locomotor parameters among rehabilitation phases were compared using one-way ANOVA followed by Tukey's HSD post hoc test. All analyses were performed in Python using standard statistical libraries.
**Clinical Guidelines:** Develop more specific clinical guidelines that could be adapted by other rehabilitation facilities facing similar challenges.	We have added a new paragraph titled *"Practical Guidelines for Dolphin Rehabilitation"* in the *Discussion* section.<br>This paragraph summarizes generalizable clinical recommendations derived from this case, outlining a two-phase rehabilitation framework that can be adapted by other facilities.<br>The new text emphasizes key clinical considerations for each phase, including wound healing, prosthesis introduction, and social reintegration, as practical guidance for managing similar rehabilitation cases.
**Visual Documentation:** Consider enhancing visual elements with additional photographs or diagrams illustrating the rehabilitation process and prosthetic design.	To address this suggestion, we have added a new supplementary video (S1 Video) showing the process of fitting the artificial tail fluke during the rehabilitation phase.<br>This visual material complements the textual description and illustrates the prosthetic design.

	Accordingly, the numbering of the other supplementary files has been updated.<br>**As this study focuses on behavioral and welfare outcomes, detailed descriptions of the prosthetic's structure and physical properties are not included here. These technical aspects will be reported separately in a forthcoming paper.**
**Future Research Directions:** Provide more specific recommendations for future research, including potential multi-institutional collaborations to address the limitations of single-case studies.	We have revised the middle part of the *Conclusions* section to incorporate this valuable suggestion.<br>The revised text now includes a more specific discussion of future research directions, emphasizing the importance of multi-institutional and collaborative approaches to overcome the limitations of single-case studies.
