# Supplementary material for: Rehabilitation outcomes following tail-fluke amputation in an Indo-Pacific bottlenose dolphin: A welfare-centered approach
Source: PLoS One. 2026 Mar 13;21(3):e0331642. doi: 10.1371/journal.pone.0331642 (PMC12987451; doi:10.1371/journal.pone.0331642)
Supplement: Attachment — Submitted filename: Response_to_the_reviewer_comments_auresp_2.docx [file pone.0331642.s010.docx]

Response to the reviewer’s comments

We would like to resubmit our manuscript entitled “Rehabilitation outcomes following tail-fluke amputation in an Indo-Pacific bottlenose dolphin: a welfare-centered approach,” which has been revised in accordance with the valuable comments provided by the reviewers and the editor. In addition to addressing each point in detail, as outlined in the response table below, we have also implemented several general revisions to enhance the overall quality of the manuscript.

| Reviewer comments | Answer comments |
| --- | --- |
| Reviewer #2 |  |
| The study would benefit from a more explicit discussion that directly highlights the advantages of your two-phase rehabilitation approach compared to other methods documented in the existing literature. | In the revised Discussion, we more explicitly emphasize the advantages of our two-phase rehabilitation framework by directly contrasting it with previously reported approaches that primarily focused on prosthetic use or short-term locomotor outcomes. Specifically, we clarify how the sequential integration of physical rehabilitation (Phase 1) and structured social reintegration (Phase 2) enabled not only functional adaptation but also sustained behavioral and social recovery, which have been less explicitly addressed in prior studies. |
| The limitation regarding the subject's maximum swim speed not returning to healthy levels should be discussed in more depth, including its implications for long-term fitness and the realistic goals of prosthetic intervention. | We expanded the Discussion to clarify that the primary goal of the prosthetic intervention was to restore species-typical locomotor patterns and swimming efficiency rather than maximum swimming speed, and to discuss the implications of this outcome for long-term fitness and welfare. |
| The reference section requires significant updating. Currently, only 11% of the citations are from the last five years (2020-2025). Incorporating recent literature is crucial to situate your work within the current state of the field. | This issue was addressed in response to the previous round of review. Following that revision, we updated the reference list through a detailed literature survey, increasing the proportion of references published within the past five years from 11% to 24%. Given the highly specialized and still limited literature on this area, the current reference list reflects the most relevant and up-to-date studies available. |
| The originality report indicates that 77% of the manuscript's content was flagged as being generated by Artificial Intelligence. This is an exceptionally high percentage that raises fundamental concerns about intellectual authenticity, authorship, and compliance with journal policy. You must provide a thorough explanation of the role of AI in the writing process and ensure that all scientific interpretation, analysis, and intellectual synthesis are unequivocally your own. | We acknowledge the concern raised by the originality report. As stated in the Acknowledgements, we used ChatGPT (OpenAI) solely for English-language editing (grammar, wording, and clarity) as non-native English-speaking authors. Importantly, AI was not used to generate or modify the scientific content of the manuscript, including the study design, data collection, statistical analyses, interpretation of results, discussion, or conclusions. All scientific decisions and intellectual synthesis were developed entirely by the authors, and the final manuscript was reviewed and approved by all co-authors. The authors take full responsibility for the content of the manuscript in accordance with journal policy. |
| Reviewer #3 |  |
| I suggest that a paragraph in discussion about the design or future improvement of the tail fluke for individuals suffering similar handicap would improve the usage time and propulsion efficiency of such prosthetic and maybe reducing the risk of infection. | We agree that prosthetic design is an important consideration for improving usage duration and propulsion efficiency. However, the present study focuses on welfare, behavioral, and functional outcomes rather than technical optimization of prosthetic structure. Detailed structural and technical aspects of the artificial tail fluke were therefore considered beyond the scope of this manuscript and will be described in a separate study. We have clarified this point in the Discussion. |
| My second suggestion is to add to the discussion of the social reintegration of the rehabilitated dolphin, whether the dolphin species has any documented information whether they take special care or consideration to handicapped individuals, or they react aggressively to individuals with unusual locomotion behavior. | Rather than adding a separate paragraph, we clarified this point within the existing Discussion by explicitly noting the limited availability of direct empirical evidence on social responses toward handicapped individuals in dolphins, thereby clearly framing our discussion and avoiding overinterpretation. |
