# Supplementary material for: Rehabilitation outcomes following tail-fluke amputation in an Indo-Pacific bottlenose dolphin: A welfare-centered approach
Source: PLoS One. 2026 Mar 13;21(3):e0331642. doi: 10.1371/journal.pone.0331642 (PMC12987451; doi:10.1371/journal.pone.0331642)
Supplement: Attachment — Submitted filename: Response to the reviewer comments.docx [file pone.0331642.s008.docx]

Response to the reviewer’s comments

We would like to resubmit our manuscript entitled “Rehabilitation outcomes following tail-fluke amputation in an Indo-Pacific bottlenose dolphin: a welfare-centered approach,” which has been revised in accordance with the valuable comments provided by the reviewers and the editor. In addition to addressing each point in detail, as outlined in the response table below, we have also implemented several general revisions to enhance the overall quality of the manuscript and to ensure full compliance with the PLOS ONE submission requirements. Specifically, the entire manuscript has been reformatted to conform to the journal’s guidelines, including adjustments to the title page structure, author affiliations, heading levels, figure and table citations, file naming conventions, and reference style.

| Reviewer comments | Answer comments |
| --- | --- |
| **Literature Review Currency:** The reference list requires substantial updating, with only 11% of references from the last five years. Incorporate recent publications (2020-2024) in marine mammal rehabilitation, prosthetics, and animal welfare science to better contextualize your work. | We have updated the reference list to include the most relevant and recent works in marine mammal welfare and rehabilitation. Consequently, the proportion of references published within the past five years has increased from about 11% to 24% (5 of 21 references). Because comprehensive rehabilitation studies in marine mammals are still at an early stage, peer-reviewed research directly addressing prosthetic-assisted rehabilitation remains scarce. This study therefore integrates the latest available evidence and provides pioneering insights that may guide future welfare-centered rehabilitation efforts. |
| **Comparative Analysis Enhancement:** While the current comparison is adequate, strengthen the discussion by comparing your findings with other marine mammal rehabilitation cases and recent advances in wildlife prosthetics. | We have expanded the Discussion to include reference to prosthetic interventions in other aquatic animals. Specifically, we mentioned studies on sea turtles that focused on the engineering and hydrodynamic design of prosthetic flippers. This addition clarifies the study’s originality by contrasting previous technically oriented attempts with the welfare-centered rehabilitation framework applied to a dolphin. |
| **Methodological Detail:** Provide more comprehensive information about assessment protocols, including specific metrics used in biologging analysis, behavioral coding systems, and inter-observer reliability measures. | We have revised the Materials and Methods section to include additional methodological details. Specifically, we added information describing the parameters recorded by the biologging devices (swim speed, stroke frequency, and tri-axial acceleration) and clarified how these variables were analyzed. In addition, we included a statement that behavioral observations were conducted objectively at fixed 5-minute intervals using scan sampling, which minimized observer bias and recording errors. These additions enhance the transparency and reproducibility of the assessment protocols. |
| **Long-term Monitoring:** Expand the discussion of long-term welfare considerations, including recommendations for ongoing monitoring and potential adaptive management strategies. | We added a new paragraph in the Discussion to address long-term welfare considerations. The revised text emphasizes the importance of continuous monitoring, adaptive management, and data sharing for sustained welfare outcomes, citing the Five Domains framework and recent reviews of marine mammal rehabilitation. |
| **Resource Implications:** Provide more detailed discussion of the practical implementation requirements, including staff training, facility needs, and cost considerations for other institutions considering similar approaches. | We have added a new paragraph in the Practical Guidelines for Dolphin Rehabilitation section to address resource and implementation considerations. The new text discusses staff training requirements, facility specifications, and cost implications, and highlights how institutional collaboration can improve feasibility and reduce expenses. |
| **Ethical Considerations:** Enhance the discussion of ethical decision-making processes throughout the rehabilitation, particularly regarding prosthesis use duration and social reintroduction timing. | We have added a new paragraph titled “Ethical Considerations in Rehabilitation” before the Evaluation of Physical Recovery and Social Behavior section. The added text describes how the duration of prosthesis use and the timing of social reintroduction were determined through medical evaluation, welfare monitoring, and behavioral assessment, emphasizing a welfare-centered and precautionary decision-making process. |
| **Statistical Analysis:** Provide clearer explanation of any statistical methods used in analyzing behavioral and locomotor data, including effect size measures where appropriate. | We have clarified the statistical analysis in the Evaluation of Swimming Efficiency Using Prosthetic Tail Flukes section. Specifically, we added a description stating that locomotor parameters among rehabilitation phases were compared using one-way ANOVA followed by Tukey’s HSD post hoc test. All analyses were performed in Python using standard statistical libraries. |
| **Clinical Guidelines:** Develop more specific clinical guidelines that could be adapted by other rehabilitation facilities facing similar challenges. | We have added a new paragraph titled “Practical Guidelines for Dolphin Rehabilitation” in the Discussion section. This paragraph summarizes generalizable clinical recommendations derived from this case, outlining a two-phase rehabilitation framework that can be adapted by other facilities. The new text emphasizes key clinical considerations for each phase, including wound healing, prosthesis introduction, and social reintegration, as practical guidance for managing similar rehabilitation cases. |
| **Visual Documentation:** Consider enhancing visual elements with additional photographs or diagrams illustrating the rehabilitation process and prosthetic design. | To address this suggestion, we have added a new supplementary video (S1 Video) showing the process of fitting the artificial tail fluke during the rehabilitation phase. This visual material complements the textual description and illustrates the prosthetic design. Accordingly, the numbering of the other supplementary files has been updated. **As this study focuses on behavioral and welfare outcomes, detailed descriptions of the prosthetic’s structure and physical properties are not included here. These technical aspects will be reported separately in a forthcoming paper.** |
| **Future Research Directions:** Provide more specific recommendations for future research, including potential multi-institutional collaborations to address the limitations of single-case studies. | We have revised the middle part of the Conclusions section to incorporate this valuable suggestion. The revised text now includes a more specific discussion of future research directions, emphasizing the importance of multi-institutional and collaborative approaches to overcome the limitations of single-case studies. |
